# Genetic markers as instrumental variables

**DOI:** 10.1016/j.jhealeco.2015.10.007

**Published:** 2016-01

**Authors:** Stephanie von Hinke, George Davey Smith, Debbie A. Lawlor, Carol Propper, Frank Windmeijer

**Affiliations:** a University of Bristol, Bristol, United Kingdom; b Imperial College London, London, United Kingdom

**Keywords:** ALSPAC, Genetic variants, Instrumental variables, Mendelian randomization, Potential outcomes

## Abstract

The use of genetic markers as instrumental variables (IV) is receiving increasing attention from economists, statisticians, epidemiologists and social scientists. Although IV is commonly used in economics, the appropriate conditions for the use of genetic variants as instruments have not been well defined. The increasing availability of biomedical data, however, makes understanding of these conditions crucial to the successful use of genotypes as instruments. We combine the econometric IV literature with that from genetic epidemiology, and discuss the biological conditions and IV assumptions within the statistical potential outcomes framework. We review this in the context of two illustrative applications.

## 1 Introduction

Many studies in the social and epidemiological sciences aim to make causal inferences using observational data. This is often problematic, as observed associations are not necessarily causal, with confounding being an important concern. Randomization of treatment, as in a Randomized Controlled Trial (RCT), is one way to infer causality. However, such experiments are not always possible or feasible. An approach commonly used in the economics and econometrics literature is that of Instrumental Variables (IV). This introduces a third variable (the instrument) that is robustly associated with the risk factor of interest, but not with the outcome variable, other than through its effect on the risk factor. This instrument can then be exploited to make causal inferences about the effect of the risk factor on the outcome.

Recently, epidemiologists, statisticians, economists and other social scientists have become interested in using genetic variants as instruments. ‘Mendelian randomization’ refers to the random assignment of an individual's genotype at conception (Davey Smith and Ebrahim, 2003, Davey Smith, 2007). Under certain assumptions that we discuss in detail below, observed associations between genetic variants and the outcome of interest are unlikely to be due to confounding by behavioural or environmental factors. Mendelian randomization can therefore be exploited to make causal inferences about the effects of modifiable (non-genetic) risk factors, on different outcomes^1^. Statisticians have highlighted some of the implicit statistical assumptions commonly made in Mendelian randomization studies (e.g. Didelez and Sheehan, 2007, Didelez et al., 2010). Genetic epidemiologists emphasize the importance of carefully examining the conditions that need to be met for genetic variants to be used as instruments (see e.g. Davey Smith and Ebrahim, 2003, Sheehan et al., 2008, Lawlor et al., 2008a, Lawlor et al., 2008b). However, while studies in economics commonly use IV methods, the (biological) conditions relevant for Mendelian randomization have not been disseminated widely in this literature. The increasing availability of biomedical information in social science datasets, however, makes understanding them crucial to the successful use of genotypes as instruments for modifiable risk factors.

The contribution of this paper is to discuss these conditions within the well-known statistical potential outcomes framework. We use the work by Imbens and Angrist (1994), Angrist et al. (1996), and Angrist et al. (2000), among others, which has been of great importance in linking the econometric IV literature to the potential outcomes framework. We link Mendelian randomization to this framework, and discuss how the conditions, defined in genetic epidemiology, relate to the IV assumptions used in statistics and economics. To communicate best practice in genetic epidemiology to a wider economics audience, we review these conditions in the context of two illustrative applications: one in social science and one in medicine. Specifically, we examine whether child fat masscausally affects (1) academic achievement, and (2) blood pressure, using 32 recently identified genetic variants as instrumental variables for fat mass.

These examples are pertinent for several reasons. For our social science application for example, obese children are more likely to be absent from school, have sleep disorders, and be treated differently by teachers, parents and peers. All these may affect children's (learning) environment and educational outcomes. However, an observed association between fat mass and academic achievement is not necessarily causal. There are likely to be many confounders, and one can never be sure that all relevant ones are accounted for. For our medical application, there is evidence that even relatively small reductions in weight can reduce blood pressure and hypertension risk (Neter et al., 2003). However, the increase in obesity in recent decades has been accompanied by a decrease in hypertension, leading to questions about their association, with some suggesting that randomized controlled trials of weight reduction could have affected blood pressure through mechanisms other than weight loss (Campos et al., 2006). The use of ordinary least squares (OLS) suggests that fat mass is inversely related to educational attainment, but increases the risk of hypertension. When using carefully selected genetic variants as instruments for fat mass, we find no evidence of a causal relationship between fat mass and academic performance, although the parameters are imprecisely estimated. In contrast, we find a positive effect of fat mass on blood pressure, suggesting that reductions in fat mass will reduce the risk of cardiovascular disease.

Although Mendelian randomization is widely used in the medical and epidemiological sciences, with its findings being fed into pharmacotherapeutic development, it is very controversial within economics. This mainly stems from the credibility of the ‘exclusion restriction’: the assumption that the variants do not directly affect the outcome of interest. Indeed, there are many situations that may violate this assumption, invalidating the instruments and biasing the estimates. One of the issues is that we have very limited knowledge and understanding of the specific functions of genes, and studies that directly examine gene-function are often underpowered. Hence, we can never be certain that the exclusion restriction is satisfied. We discuss this in detail, and highlight the specific (biological) pathways through which the use of genetic variants as instrumental variables may lead to invalid inferences, including the potential for variants to have multiple functions, or to be correlated to other variants that affect the outcome of interest. We also consider the implications of gene–gene and gene–environment interactions for Mendelian randomization. Finally, it is worth noting that the uncertainty of the exclusion restriction is not specific to Mendelian randomization. Indeed, any IV analysis relies on this untestable assumption, and one generally assesses such studies based on whether the available evidence suggests that the assumption is likely to hold (see also von Hinke et al., 2012). We discuss different ways of exploring its validity indirectly in the context of Mendelian randomization and attempt to clearly articulate the potential situations that would invalidate the approach^2^.

Section 2 details the conditions that need to be met for genetic variants to be used as instruments. Section 3 introduces our empirical application. We describe the data, examine the validity of our choice of genetic variants, present the results as well asa number of sensitivity checks. Section 4 concludes and discusses the implications of our findings in terms of best practice for using Mendelian randomization by researchers who do not come from a primarily biological discipline.

## 2 The use of genetic variants as instrumental variables

We start by discussing the links between Mendelian randomization and other approaches used in the medical and social science literature. We then build on the Potential Outcomes Framework by Imbens and Angrist (1994), Angrist and Imbens (1995), Angrist et al., 1996, Angrist et al., 2000. We first briefly outline the well-known structural assumptions in the context of our applications, and then discuss how Mendelian randomization links to the statistical assumptions of this framework.

### 2.1 Mendelian randomization

We discuss Mendelian randomization from a statistics and economics perspective in the context of a social study, with the aim of making causal inferences about the effect of a treatment on an outcome of interest. Depending on the discipline, the terms ‘treatment’, ‘risk factor’, ‘exposure’, ‘predictor’, or ‘intermediate phenotype’ have all been used to denote the variable of interest that potentially causes the outcome. To avoid confusion, the remainder of this paper uses either the term ‘treatment’ or ‘risk factor’.

The concept of Mendelian randomization is closely linked to Randomized Controlled Trials (RCTs), where the allocation of treatment is randomized over all eligible individuals (Davey Smith and Ebrahim, 2005, Hingorani and Humphries, 2005). Indeed, IV can be applied to analyse encouragement designs (such as RCTs where the instrument is the encouragement to participate) that are affected by non-ignorable non-compliance. Non-compliance refers to the fact that individuals can choose to take or not take treatments other than those to which they are randomized. Non-ignorable non-compliance refers to participants choosing to take or not to take the treatment that they are randomized to in a manner associated with their study outcomes, after adjusting for baseline characteristics. This is also known as endogenous treatment in economics, or selection into treatment.

The idea is similar for the social context in our application: individuals ‘select’ their treatment – fat mass – through lifestyle choices, such as diet and physical activity, which are likely to be related to their study outcome (educational attainment and blood pressure). In a well-conducted RCT of an intervention aimed at reducing fat mass, the random allocation effectively balances these lifestyle choices between groups. Comparing groups based on the original random allocation (‘intention to treat’) maintains this balance, whereas comparing groups based on what treatment was actually chosen by the participant (a ‘per-protocol’ analysis) is likely to be biased due to non-ignorable non-compliance. In other words, treatment by choice (as opposed to treatment by randomization) is likely to be related to the outcome through characteristics such as social class, income, diet, etc.

There are many cases, however, where RCTs are infeasible (for example, there may be no effective intervention to randomize, such as for adiposity) or unethical (for example, when examining the effect of prenatal alcohol consumption on different outcomes). In such cases, quasi-experimental designs such as Mendelian randomization experiments can provide a useful alternative approach.

As in RCTs, Mendelian randomization assumes that characteristics such as social class, income and diet are balanced across the genotypes. This assumption exploits the fact that there is an equal probability that either parental allele (see Appendix A) is transmitted to offspring. As this allocation is random at the family trio level, the cleanest experiment is one with biological siblings, examining randomization of genes within families (Davey Smith and Ebrahim, 2003). However, also at a population level, many studies suggest that genetic variants are largely unrelated to the many socioeconomic and behavioural characteristics that are closely linked with each other and that confound conventional observational studies (Bhatti et al., 2005, Davey Smith et al., 2008, Kivimäki et al., 2008, Lawlor et al., 2008a, Lawlor et al., 2008b, von Hinke et al., 2014; see also Fisher, 1952; Box 2010). Hence, as genes are randomly assigned during meiosis (cell division for reproduction), individuals of different genotypes are expected not to differ systematically in any other respect. The issue of compliance in Mendelian randomization studies is discussed further in Section 2.2.

Interventional studies such as RCTs, where treatment is introduced at a certain age, identify the effect of differences in treatment from that point in time. By contrast, estimates from Mendelian randomization experiments exploit differences in treatment throughout life, estimating the long term (cumulative) effects of the treatment on the outcome of interest (Davey Smith and Ebrahim, 2005).

As noted by Didelez and Sheehan (2007), the potential limitations of Mendelian randomization studiesfall into two sets. First, limitations related to the implicit statistical assumptions common in many Mendelian randomization studies, such as linearity and additivity. As these have been discussed in detail (e.g. Didelez and Sheehan, 2007, Didelez et al., 2010), we focus on the second set of limitations: those relating to the assumptions of the validity of the instrument. Genetic epidemiology studies emphasize the importance of carefully examining several situations and (biological) processes that may violate the IV assumptions (see e.g. Davey Smith and Ebrahim, 2003, Sheehan et al., 2008, Lawlor et al., 2008a, Lawlor et al., 2008b). The increasing availability of biomedical information in social science datasets makes understanding of these conditions crucial to the successful use of genotypes as instruments for modifiable risk factors. We therefore first outline the structural assumptions in the context of our applications, discuss the concepts defined in epidemiology and relate them to the assumptions that need to be met to obtain causal estimates of the effect of the risk factor on the outcome of interest, as defined in the statistics and economics literature.

### 2.2 The potential outcomes framework

Our two illustrative applications use a continuous treatment and outcome variable, and 32 independent genetic variants. For ease of exposition, however, we discuss our framework for the case of two genetic variants, though we note that this is easily generalized to incorporate any number of variants. As shown in Angrist et al. (2000), with such discrete instruments, the number of instruments is irrelevant; it is the number of distinct values of the instrument vector that matters. Hence, with two genetic variants (denoted by *Z*_1_ and *Z*_2_), we observe nine instrumental values, defined by the combination of the number of rare alleles. Without loss of generality, we order the instruments by their mean fat mass, for example: $\left(Z_{1},Z_{2}\right)\in \left\{\left(0,0\right),\left(0,1\right),\left(1,0\right),\left(0,2\right)\left(1,1\right),\left(1,2\right),\left(2,0\right),\left(2,1\right),\left(2,2\right)\right\}$. We can represent this as an instrument *Z* with support 1,2,…,9; the full set of instruments are the nine mutually exclusive dummy variables. Let *A* and *Y* denote random variables representing, respectively, fat mass and the educational outcome/blood pressure.

Let $A_{i}\left(z\right)$ be the potential fat mass for individual *i* when the instrument is set to *z*. Only one of the 9 possible treatment assignments $A_{i}\left(1\right),A_{i}\left(2\right),\ldots ,A_{i}\left(9\right)$ is ever observed for any one individual. Similarly, let $Y_{i}\left(z,a\right)$ be the potential outcome for individual *i* that would be obtained if *i*‘s fat mass, the treatment variable, was set to *a* and the instrument was set to *z*. We refer to $A_{i}\left(z\right)$ and $Y_{i}\left(z,a\right)$ as the potential treatments and potential outcomes respectively. Similar to $A_{i}\left(z\right)$, only one of the 9 potential outcomes $Y_{i}\left(1,A_{i}\left(1\right)\right),Y_{i}\left(2,A_{i}\left(2\right)\right),\ldots ,Y_{i}\left(9,A_{i}\left(9\right)\right)$ can ever be observed for any one individual.

We follow convention and assume that individual *i*'s potential treatments and potential outcomes are independent of the outcome and treatment status of other individuals. This is also referred to as the Stable Unit Treatment Value Assumption (SUTVA, see e.g. Rubin, 1980).

Given the set of potential outcomes, we can define the causal effects for individual *i* of *Z* on *A* as $\left(A_{i}\left(z\right)-A_{i}\left(z-1\right)\right)$, and the causal effects for individual *i* of *Z* on *Y* as $\left(Y_{i}\left(z,A_{i}\left(z\right)\right)-Y_{i}\left(z-1,A_{i}\left(z-1\right)\right)\right)$, for $z\in \left\{2,\ldots ,9\right\}$. These are also known as the intention-to-treat effects.

Assumption 1 **Independence**

$$Z_{i}╨{\left\{Y_{i}\left(z,a\right),A_{i}\left(z\right)\right\}}_{z,a}$$

The independence assumption implies that the instrument is independent of all potential outcomes and potential treatments, for all values of *z* and *a*. In other words, the instrument is as good as randomly assigned. Note that, as Mendelian randomization is closely related to RCTs, where the allocation of treatment is randomized over all eligible individuals, we specify the *unconditional* independence assumption. If the instruments are not independent of covariates, however, we require the *conditional* independence assumption, implying that independence holds conditional on some vector of covariates, defined by **X**. We discuss the role of covariates in Mendelian randomization studies in more detail in Section 2.4.

Given SUTVA and independence, we can obtain unbiased estimators for the average intention-to-treat effects by taking the difference of sample averagesof the outcomes and treatments at different values of the instrument.

Assumption 2 **Exclusion**

$$Y_{i}\left(z,a\right)=Y_{i}\left(z',a\right),\;\text{for}\;\text{all}\;z,z^{\prime }\;\text{and}\;\text{for}\;\text{all}\;a\text{.}$$

This implies that $Y_{i}\left(1,a\right)=Y_{i}\left(2,a\right)=\ldots =Y_{i}\left(9,a\right)$, for all *a*, or that the potential outcomes, at any level of fat mass *a*, are unchanged by the value of the instrument. In other words, the only way through which the instrument affects the potential outcome is via *A*. It implies that $Y_{i}\left(z,a\right)$ is a function of *a* only, and hence we can write $Y_{i}\left(z,a\right)=Y_{i}\left(a\right)\text{.}$ If the exclusion restriction only holds conditional on **X**, we can specify the exclusion restriction conditional on these covariates.

With heterogeneous responses, the potential outcomes for individual *i* can be written as a general function of *a*, say $Y_{i}\left(a\right)\equiv g_{i}\left(a\right)\text{.}$ We can define the individual causal effects of *A* on *Y* as the derivatives of $g_{i}\left(a\right)\text{.}$ So the individual causal response is the difference in potential outcomes at each value of *a*.

Although we can never observe any of these individual causal effects, we can observe the *average* causal effects for groups of individuals who can be induced to change treatment: $E\left[{g^{\prime }}_{i}\left(q\right)|A_{i}\left(z\right)<q<A_{i}\left(z^{\prime }\right)\right]$, where ${g^{\prime }}_{i}\left(q\right)$ is the derivative of $g_{i}\left(a\right)$ w.r.t. *a* evaluated at *q*. Inferences about such average causal effects are made using changes in treatment status that are induced by the instrument (Angrist et al., 1996). For this, we require the instrument to affect treatment status.

Assumption 3 **Non-zero effect of the instrument on treatment**

$$E\left[A_{i}\left(z\right)-A_{i}\left(z'\right)\right]\neq 0,\;\text{for}\;\text{all}\;z,z^{\prime }$$

This implies that expected potential fat mass is affected by the instrument and therefore that the instrument has an effect on the treatment.

Assumption 4 **Monotonicity**

$$\begin{array}{l} P\left[A_{i}\left(z\right)\geq A_{i}\left(z^{\prime }\right)\right]=1\;\text{for}\;\text{all}\;z\geq z^{\prime }\;\text{or}\\ P\left[A_{i}\left(z\right)\leq A_{i}\left(z^{\prime }\right)\right]=1,\;\text{for}\;\text{all}\;z\leq z^{\prime }, \end{array}$$

for all *i*. This implies that the potential fat mass for individual *i* with instrument value *z* is at least as high as the potential fat mass for the same individual with instrument value *z*′, or vice versa, that the potential fat mass for individual *i* with instrument value *z* is lower than or equal to the potential fat mass for the same individual with instrument value *z*′, for all *i*.

We use the above assumptions to interpret differences in average outcomes and treatments at different values of the instrument. Under these assumptions, the IV estimand is equal to a weighted average of the 8 linearly independent average causal responses (ACRs), defined here as *τ*_*z*,*z*−1_:

$$\begin{array}{l} \tau_{z,z-1}=\frac{E\left[Y_{i}|Z_{i}=z\right]-E\left[Y_{i}|Z_{i}=z-1\right]}{E\left[A_{i}|Z_{i}=z\right]-E\left[A_{i}|Z_{i}=z-1\right]}\\ =\frac{\int E\left[{g^{\prime }}_{i}\left(q\right)|A_{i}\left(z-1\right)<q<A_{i}\left(z\right)\right]P\left\{A_{i}\left(z-1\right)<q<A_{i}\left(z\right)\right\}\text{d}q}{\int P\left\{A_{i}\left(z-1\right)<q<A_{i}\left(z\right)\right\}\text{d}q},\;\text{for}\;z=2,\ldots ,9. \end{array}$$

In other words, each instrumental variable identifies a unique causal parameter, one specific to the subpopulation whose treatment is affected by the instrument. Different valid instruments may thereforelead to different causal parameters. Hence, IV estimation that uses each of the instruments one by one weights the derivative function ${g^{\prime }}_{i}\left(q\right)$ by the strength of the instrument (Angrist et al., 2000, Angrist and Pischke, 2009). Let the points of support of *Z* be ordered such that *l* < *m* implies that $E\left[A_{i}|Z_{i}=l\right]<E\left[A_{i}|Z_{i}=m\right]\text{.}$ The IV estimand using all 9 mutually exclusive instruments can then be written as:

(1) $$\overset{9}{\underset{z=2}{\sum }}\omega_{z}\tau_{z,z-1}$$

where

$$\omega_{z}=\left(E\left[A_{i}|Z_{i}=z\right]-E\left[A_{i}|Z_{i}=z-1\right]\right)\cdot \frac{\sum_{l=z}^{9}\pi_{l}\left(E\left[A_{i}|Z_{i}=l\right]-E\left[A_{i}\right]\right)}{\sum_{l=1}^{9}\pi_{l}E\left[A_{i}|Z_{i}=l\right]\left(E\left[A_{i}|Z_{i}=l\right]-E\left[A_{i}\right]\right)}$$

and $\pi_{l}=\mathrm{Pr}\left[Z=l\right]$, *ω*_*z*_ > 0 and ∑_*z*_*ω*_*z*_ = 1. Hence, it averages the (pairwise) instrument-specific weighted averages of the derivative function, where the weights are proportional to the instrument-induced change in the cumulative distribution function (CDF) of fat mass (Angrist and Imbens, 1995, Angrist et al., 2000, Angrist and Pischke, 2009). In other words, it combines a set of weighted average effects into a new weighted average.

The above example is for a case with two genetic variants, or nine instrumental variables, which is straightforward to estimate in standard software packages. As discussed earlier, however, we observe 32 genetic variants, and with that, a maximum of 3^32^ instrumental variables^3^. Instead of specifying a model with that many instruments, we use one ‘allelic score’, mapping the instrumental variables *z* = 1, …, 3^32^ onto a function $h\left(Z_{i}\right)\text{,}$ defined as the number of risk alleles carried by each child. In other words, for every individual, we sum the number of adiposity-increasing alleles for the 32 variants. For example, the allelic score for the above case of 9 instruments takes five values:

**Table:** 

$\left(Z_{1},Z_{2}\right)$	$h\left(Z_{i}\right)$
$\left(0,0\right)$	0
$\left(0,1\right),\left(1,0\right)$	1
$\left(1,1\right),\left(0,2\right),\left(2,0\right)$	2
$\left(1,2\right),\left(2,1\right)$	3
$\left(2,2\right)$	4

(in our sample, the allelic score using the 32 genetic variants takes one of 28 distinct values, see also Section 3.4). Using the allelic score $h\left(Z_{i}\right)$, the IV estimand can be written as:

$$\frac{\text{Cov}\left(Y_{i},h\left(Z_{i}\right)\right)}{\text{Cov}\left(A_{i},h\left(Z_{i}\right)\right)}\text{.}$$

Similar to (1), the IV estimand that uses the multivalued allelic score $h\left(Z_{i}\right)$ is a weighted average of the ACRs *τ*_*z*,*z*−1_. However, the weights are now given by:

$$\omega_{z}=\frac{\left(E\left[A_{i}|Z_{i}=z\right]-E\left[A_{i}|Z_{i}=z-1\right]\right)\cdot \sum_{l=z}^{9}\pi_{l}\left(h\left(Z_{i}=l\right)-E\left[h\left(Z_{i}\right)\right]\right)}{\sum_{m=1}^{9}\left(E\left[A_{i}|Z_{i}=m\right]-E\left[A_{i}|Z_{i}=m-1\right]\right)\cdot \sum_{l=m}^{9}\pi_{l}\left(h\left(Z_{i}=l\right)-E\left[h\left(Z_{i}\right)\right]\right)}\text{,}$$

where, again, $\pi_{l}=\mathrm{Pr}\left[Z=l\right]$, *ω*_*z*_ > 0 and ∑_*z*_*ω*_*z*_ = 1 As above, the weights are proportional to the first stage impact on the treatment (Angrist et al., 2000). Thus, inferences about the ACRs are made using changes in treatment status that are induced by the instrument.

At this point, it may be useful to consider a situation where the instrument and treatment are binary, in which case we can stratify individuals into four latent groups, as commonly used in the econometrics literature (e.g. Angrist et al., 1996): those who are induced to take treatment by the instrument (*compliers*), those who do the opposite of their assignment (*defiers*), those who never take treatment, whatever their assignment (*never-takers*), and those who always take treatment, regardless of their assignment (*always-takers*). In this framework, the IV estimate can be interpreted as a Local Average Treatment Effect (LATE) or Complier Average Causal Effect (CACE): the effect of treatment for those who are induced to take treatmentby the instrument (the compliers). Indeed, LATE or CACE is not informative about effects on never-and always-takers, because (by definition) treatment status for these groups is not affected by the instrument. By virtue of the exclusion restriction, the causal effect of *Z* on *Y* for never-takers and always-takers is zero. And by virtue of the monotonicity assumption, there are no defiers. Note that in the case of a continuous intermediate variable as generally used in Mendelian randomization studies (such as fat mass, lipids, energy intake, units of alcohol, number of cigarettes, etc.), it is unclear what it means to be a complier, defier, always-taker, or never-taker. A detailed discussion on identifying compliers in such cases is beyond the scope of this paper, but for different approaches, we refer the reader to Frangakis and Rubin (2002), Sjölander et al. (2009), Jin and Rubin (2008), and Bartolucci and Grilli (2011).

### 2.3 Conditions for the use of genetic variants as instrumental variables

For a valid causal interpretation of the IV estimand that uses the genetic variants as instruments, we require the above assumptions to hold. However, there are various situations that may violate them, which need to be examined. We discuss these below.

Assumption 1 **Independence**

Although genotypes are randomly allocated at conception, the allele distribution may differ for different population subgroups. If these subgroups also have systematically different outcomes of interest, this could lead to an association between the two at the population level without an actual causal relationship. A systematic relationship between the allele frequency and the outcome of interest across different sub-populations is also referred to as ‘population stratification’. For example, allele frequencies can vary across ethnic groups. Any systematic differences in the outcome of interest across these subpopulations that are not due to the genetic make-up may therefore lead to biased estimates of the effect of treatment by violating the independence Assumption 1. In other words, despite the fact that genotypes are randomly allocated and with that satisfy independence, any population stratification can violate this assumption. This can be dealt with, however, by examining the question of interest *within* ethnic groups, separately analyzing the different subpopulations, and/or adjusting for principal components from genome wide data that function as ancestry markers. These approaches then relyon the *conditional* independence assumption, assuming independence *conditional* on ethnicity or ancestry.

As genotypes are randomly assigned *given* the parental genes, the presence of assortative mating based on genes can violate independence. The cleanest experiment, therefore, is one with biological siblings, examining randomization of genes *within* families (Davey Smith and Ebrahim, 2003). However, even when we only observe one individual per family, Mendelian randomization is valid if we are able to assume that, at the population level, genetic variants are unrelated to other characteristics that may affect the outcome of interest, as shown in many studies (Bhatti et al., 2005, Davey Smith et al., 2008, Kivimäki et al., 2008, Lawlor et al., 2008a, Lawlor et al., 2008b, von Hinke et al., 2014; see also Fisher, 1952). One way to indirectly test independence is by exploring whether the distribution of observable characteristics is the same in different groups defined by the value of the instrument (i.e. different genotypes). Indeed, if the instrument is randomized, there should be no systematic variation in the covariates by genotype, whether we use a within- or between-family analysis. This raises the question however, about *which* covariates to test for, as any characteristic is, in principle, a post-treatment variable with respect to the instrument. Hence, any systematic variation in these indirect tests does not necessarily indicate a violation of independence (see also Rubin, 2005). It may be, for example, that the instrument is picking up other causal effects of the same treatment.

Assumption 2 **Exclusion**

One can never directly test whether exclusion holds, and there are various situations in which Assumption 2 fails, invalidating the instruments. One such situation is that ‘behaviours’ may be affected by the genotype. As individuals inherit their genes from their parents, it may be important to consider whether parents’ behaviours or preferences are affected by their genotype (and hence their offspring's genotype). This can bias studies that examine maternal behaviours that influence the outcome of interest via intrauterine effects. Likewise, it may be a problem in studies where parental behaviours influence the outcomevia affecting their (child's) behaviour.

As an example of the former, if one were interested in the effect of an individual's alcohol consumption on their later life liver disease using a genetic variant that robustly relates to alcohol intake, any intrauterine effects of maternal alcohol consumption during pregnancy on offspring liver development could violate Assumption 2. This is because the mother's genotype is related to the offspring's genotype (the instrument), and will influence her alcohol consumption throughout life, including potentially when she was pregnant. If maternal alcohol intake during pregnancy affects her offspring's liver development *in utero* and its functioning later in the child's life, there is a link from the offspring's genotype (IV) to the outcome (the offspring's liver disease) via maternal genotype and maternal alcohol consumption, violating Assumption 2.

As an example of the latter, if one is interested in the effect of fat mass on education, as we are below, parents who carry ‘fat’ alleles may be discriminated against in the labour market because of their on average higher weights (Cawley, 2004). If this affects their behaviour or preferences for their child's weight or education, Assumption 2 may be violated.

A second situation relates to the mechanisms through which genetic variants affect the modifiable risk factor. These are often unknown. If the mechanism involves changes in behaviour or preferences that in addition to affecting the risk factor also directly affect the outcome, Assumption 2 will be violated. For example, if the fat related genetic variants that we use below influence fat mass because they are related to pathways associated with addiction more generally, such as addiction to high energy foods, and if the latter affects the outcome of interest, Assumption 2 would be violated. If the mechanism only results in changes to the risk factor but does not directly affect the outcome, the exclusion restriction is not violated.

Thirdly, the genetic instrument may be related to other genetic variants that affect the outcome of interest. Mendel's second law states that the inheritance of one trait is independent of the inheritance of another. However, it has been shown that this does not always hold and that some variants are likely to be co-inherited. This so-called ‘Linkage Disequilibrium’ (LD) does not occur for genetic variants on different (non-homologous) chromosomes, and the degree of LD is partly a function of the distance between the loci (see Appendix A for some of the genetic terms used here). Depending on the effects of the co-inherited variant, LD can bias the estimates. If our instrument is in LD with another polymorphic locus that affects only the modifiable risk factor, the IV estimates remain consistent. However, if it is in LD with a polymorphic locus that directly affects the outcome, Assumption 2 is violated. Relatedly, there is the situation of ‘pleiotropy’, where one genetic variant has multiple functions. The case is similar to that of LD, and will invalidate the IV approach if the pleiotropic effect influences the outcome directly, but not if it affects only other characteristics that are unrelated to the outcome of interest.

Fourth, a biological process that may bias causal estimates in Mendelian randomization studies is ‘canalization’. This refers to the reduced sensitivity of a phenotype to the changes in underlying genetic and non-genetic factors that determine its expression. Hence, a canalized genotype produces the same (or a similar) phenotype in different genetic and non-genetic backgrounds (Flatt, 2005). For example, an individual who has a genetic variant associated with higher blood pressure may not experience adverse phenotypic effects of high blood pressure due to the arteries becoming resistant. This is difficult to test for, as the genetic variant may still be related to blood pressure, but any adverse health outcomes (phenotypes) normally caused by higher blood pressure would not occur. Hence, canalization implies that the genotype can affect the outcome through alternative channels, altering the association between genotype and outcome, without any change in the genotype-risk factor relationship. Canalization, therefore, canviolate the exclusion restriction.

These different potential violations of Assumption 2 indicate that, as the specific functions of genetic variants and the mechanisms through which they affect individuals are often unknown, one can never claim that exclusion holds. Indeed, one can only test exclusion *indirectly*; we discuss this in more detail in Section 2.5 below.

Assumption 3 **Non-zero effect of the instrument on treatment**

A valid instrument must be associated with (i.e. have a non-zero effect on) the risk factor of interest. Mendelian randomization can only be used with genetic variants that have been robustly shown to affect the risk factor. This point is especially important, as many initial genotype-risk factor associations fail to replicate (Colhoun et al., 2003). Without a consistent population association, even if a *sample* correlation exists, Assumption 3 may be violated. Indeed, choosing SNPs merely based on the *sample* association with the risk factor (rather than using information external to the study) can lead to biased IV estimates (Taylor et al., 2014). It is therefore important that Mendelian randomization studies only use genetic variants that have been shown to be robustly associated with the risk factor in a large number of independent studies.

However, even if a suitable genetic instrument is available, it may explain little of the variation in the observed risk factor. A weak association could result in a biased IV estimate and has implications for statistical power. If the alleles shift the distribution of risk factor by a very small amount, the effect of the risk factor on the outcome is identified only by this small difference, emphasizing the need either for very large sample sizes, especially when the average causal effect of the risk factor on the outcome could be small, or many genetic variants that can be combined into a more powerful instrument. This, of course, is not a problem specific to Mendelian randomization, but refers to a more general problem of weak instruments (see e.g. Bound et al., 1995, Staiger and Stock, 1997, Stock et al., 2002).

Assumption 4 **Monotonicity**

Whether monotonicity is satisfied relies on knowing each individual's counterfactual and therefore always remains an assumption. Monotonicity may be violated in the presence of gene–environment interactions; i.e. when the effect of the environment on the risk factor differs depending on individuals’ genetic predisposition, or when individuals’ genetic predispositions are expressed differently in different environments. For example, if the expression of a genetic variant that increases fat mass depends on individuals’ awareness of the importance of nutrition for one's health, monotonicity may be violated when the potential fat mass of an individual with the genetic variant in an (e.g. educated) environment is less than the potential fat mass for the same individual in that environment without the genetic variant. However, if the expression of the variant is simply reduced in the educated environment, monotonicity would not be violated, as the potential adiposity of an individual with the genetic variant remains at least as high as the potential adiposity for the same individual without the genetic variant.

### 2.4 The role of covariates in mendelian randomization experiments

There is a large literature on the use of covariates in IV. Economics and social applications of IV generally include a wide set of control variables; the main motivation being that the *conditional* independence and exclusion restriction are more likely to be valid. A second reason for including covariates in many economics and social applications of IV is that it may reduce the residual variability of the dependent variable, leading to more precise estimates.

The situation is somewhat different, however, in RCTs/encouragement designs, and in Mendelian randomization studies. When covariates enter the assignment mechanism in RCTs/encouragement designs, such as when randomization takes place within certain strata, these covariates should be controlled for, relying on the *conditional* independence assumption. The inclusion of further baseline covariates may in addition increase the precision of the estimates. In Mendelian randomization studies, however, there are no baseline covariates. Furthermore, as covariates do not enter the assignment mechanism, assignment is independent of covariates, and we can rely on the unconditional independence assumption. Therefore, conditioning on covariates is not necessary in Mendelian randomization experiments. The exception, however, is when there is population stratification. In this case, the analyses should be done *within* population subgroups, or should adjust for principal components from genome wide data, relying on the *conditional* independence assumption.

Although one may choose to adjust for covariates to increase precision, this raises the issue of *which* covariates to include in a Mendelian randomization study, as any characteristic is, in principle, a post-treatment variable with respect to the instrument, and – with that – may be affected by the instrument. If the instrumented treatment has multiple causal effects, or if the outcome has a causal effect of its own, adjusting for such post-treatment variables may lead to biased estimates of the causal effect. Indeed, we should not control for any ‘downstream’ (behavioural) covariates that are potentially affected by the treatment or outcome. Thus, we only control for 10 ancestry-informative principal components in our main analyses, though we report the estimates that adjust for further covariates in the sensitivity analysis. Under independence, and when the instrumented treatment and outcome do not affect these covariates, the unadjusted and adjusted IV estimates should be similar, though the latter may be more precise.

### 2.5 Testing the exclusion restriction

There is no *direct* test for the validity of the exclusion restriction (Assumption 2). In other words, its validity will never be known with certainty and can only be examined indirectly or falsified by the data. To this end, however, Mendelian Randomization is no different from any other (non-genetic) IV study; the exclusion restriction *always* remains an assumption. However, one of the differences, one may argue, is that the specific functions of genetic variants and the mechanisms through which they affect individuals are often unknown, making it more difficult, if not impossible, to argue that the instrument is valid. However, with a rapidly growing medical literature, our knowledge on the specific function of variants is increasing. For example, much is now known about the function of certain variants in the metabolism of alcohol, leading to clear predictions from the medical literature on how they affect individuals’ alcohol consumption. But knowing the exact function of a variant is not sufficient. Indeed, the variant may be pleiotropic, and its pleiotropic effects may have not yet have been identified, potentially invalidating the instrument.

Similar to other IV studies that use multiple instruments, however, Mendelian randomization allows for potential violations through pleiotropy or LD (though not necessarily canalization) to be tested when data is available on a large number of genetic instruments. More specifically, if multiple IV models – each using different combinations of these variants – predict the same causal effect, it is unlikely to be due to some common pleiotropy or LD across the different sets of variants, assuming that the different variants are located on different chromosomes and affect the trait via different pathways (Davey Smith, 2011, Palmer et al., 2011, von Hinke et al., 2013). Hence, consistency between such estimates provides evidence *against* potential pleiotropy or LD-induced confounding. However, obtaining different causal effects with different combinations of variants does not *necessarily* point to a violation of the exclusion restriction, as variability in treatment effects may occur due to different compliant subpopulations for the different instrument sets (i.e. different LATEs or CACEs).

Alternatively, one can view Mendelian randomization with multiple instruments as analogous to a meta-analysis of separate study results. Just like the IV estimate using multiple instrumental variables is a weighted average of the individual IV estimates, a meta-analysis is a weighted average of multiple studies. Bowden et al. (2015) show that, in this setting, Egger regression (a tool to detect small study bias in meta-analysis) can be adapted to test for bias from pleiotropy. In addition, it can provide an estimate of the causal effect of the treatment on the outcome of interest, even when the genetic variants are invalid.

In a constant effects model, one can indirectly test whether the exclusion restriction holds using an ‘over-identification’ (Sargan or Hansen) test (Sargan, 1958, Hansen, 1982), provided that there are more instruments than endogenous variables. Note, however, that this is not a test that the instruments are indeed valid. A problem with the over-identification test is that it has low power, especially when the underlying IV estimates are imprecise. In our heterogeneous treatment effects framework however, over-identification tests are inappropriate, even when the underlying estimates are precise, as a rejection of the test need not imply a violation of the exclusion restriction. As discussed above, it may point to treatment effect heterogeneity, as different valid instruments may estimate different parameters, with the final IV estimate being a weighted average of the different treatment effects. Hence, although we report the test statistic below, we cannot necessarily interpret it in a heterogeneous treatment effects framework.

Note that, although canalization refers to a violation of the exclusion restriction, it cannot necessarily be tested using over-identification tests. Similar to the above, a rejection cannot distinguish between treatment effect heterogeneity and canalization. In fact, there is no (clear) way of testing or correcting for canalization. However, for the complex traits that are largely of interest in Mendelian randomization studies, there is no evidence that canalization occurs in humans (Davey Smith and Ebrahim, 2003).

### 2.6 Previous studies in the economics literature

The existing economics literature includes three studies that exploit genetic variation to identify the effects of BMIon economic outcomes. Ding et al. (2009) examine the effects of several health conditions, one of which is BMI, on adolescents’ academic achievement. Their IV results show large and significant negative effects on girls’ Grade Point Average (GPA), but not for boys. GPAs for obese girls are on average 0.8 points lower than for non-obese girls. They use four genetic variants as instruments: the dopamine transporter (*DAT1*) and D2 receptor (*DRD2*), tryptophan hydroxylase (*TPH*) and cytochrome P4502B6 (*CYP2B6*). Fletcher and Lehrer (2011) take a similar approach to Ding et al., but use the Add Health data to exploit within-family genetic inheritance. They find no evidence that obesity affects academic achievement. In addition to *DAT1* and *DRD2*, their instruments include the dopamine D4 receptor (*DRD4*), the serotonin transporter (*5HTT*), monoamine oxidase (*MAOA*) and cytochrome P4502A6 (*CYP2A6*). Finally, Norton and Han (2008) examine the effects of BMI on labour market outcomes using *DAT1* and *DRD4* as instruments for BMI and find no evidence of a causal association.

The discussion in Section 2.3 above highlights the importance of the choice of genetic variants in Mendelian randomization experiments. Although the validity of the exclusion restriction can never be tested directly, it is unlikely that genes related to neurotransmitters such as dopamine receptors and serotonin transporters are valid instruments. The inherent problem is that neurotransmitters are implicated in many different neurological processes. Hence, it is difficult to argue that they can be used as valid instruments for one specific risk factor without being associated with others that could plausibly influence the outcome of interest (Cawley et al., 2011, von Hinke et al., 2011).

More generally, however, Mendelian randomization can only be used with genetic variants that have been robustly shown to affect the risk factor (Assumption 4), relying on *prior* knowledge about the association between the genotype and risk factor. The choice of instruments in the studies cited above, however, seems to be data-derived: using either forward stepwise estimation (Ding et al.) or selecting those SNPs that have nominally statistically significant *sample* correlations in the first stage (Fletcher and Lehrer). Furthermore, both Ding et al. and Fletcher and Lehrer acknowledge that there is weak and inconsistent evidence in the medical literature, based on very small unrepresentative clinical samples, of the association between their genetic variants and health status or behaviours. Indeed, the IV strategy is invalid when relying only on such *sample* associationsand leads to biased results (Taylor et al., 2014). Norton and Han (2008) base their selection of SNPs on a study by Guo et al. (2006), who find a negative association between the D4.7/D4.7 genotype of *DRD4* and obesity. This relationship, however, has not been replicated in other independent studies (see for example Hinney et al. (1999), or Fletcher and Lehrer (2011) who find an insignificant but *positive* association; see also Lawlor et al., 2008a).

To our knowledge, there are no studies in economics that explore the effects of BMI on blood pressure. However, the relationship has been explored in the medical literature. Indeed, Timpson et al. (2009) use two genetic variants, *FTO* and *MC4R*, as instruments for BMI to explore the effects on blood pressure and hypertension risk. Using those aged 20 and over from the Copenhagen General Population Study, they find that BMI increases both systolic and diastolic blood pressure. We are, however, not aware of any studies exploring this relationship for children or adolescents.

## 3 Application: The effect of fat mass on academic performance and blood pressure

### 3.1 Data

Our data are from a cohort of children born in one geographic area (Avon) of England. Women eligible for enrolment in the population-based Avon Longitudinal Study of Parents and Children (ALSPAC) had an expected delivery date between 1 April 1991 and 31 December 1992. Approximately 85% of these mothers enrolled, leading to about 14,000 pregnancies. The Avon area has approximately 1 million inhabitants and is broadly representative of the UK as a whole, though slightly more affluent than the general population^4^. Detailed information on the study children and their families has been collected using a variety of sources, including self-completed questionnaires, data extraction from medical and educational records, in-depth interviews, and biological samples. Note however, that ALSPAC is a cohort; as such, there is no systematic data collection on siblings.

A total of 12,620 children survived past the age of 1 and returned at least one questionnaire. Of these, we exclude 642 children because either their mother or father is of non-white ethnic origin (to avoid potential population stratification), leaving 11,978 potential participants. Our sample selection process is as follows. First, we select children for whom we observe their genotypes and 10 ancestry-informative principal components, leaving us with 7335 children. Second, we drop children with missing data on fat mass. We further restrict the sample to children for whom we observe their educational outcomes and blood pressure, leading to a final sample size of 4844 and 4047 children, respectively.

Attrition in the ALSPAC cohort is known to be correlated with socio-economic position, with children from lower educated, lower income families more likely to drop out (Boyd et al., 2012, Fraser et al., 2012). Attrition can bias our analyses if observations are lost in a non-random manner. We explore attrition more generally in Table 1. Column 1 shows the descriptive statistics for the full sample of children for whom data is available. Column 2 shows the statistics for the sample with genetic information, and column 3 and 4 use the final estimation sample for educational outcomes and blood pressure respectively. We confirm the socio-economic gradient in attrition: children in the estimation sample do significantly better in school, based on their Key Stage 3 (KS3) tests, their families are wealthier than the original sample, of higher socio-economic position, and their mother are higher educated and older, with fewer mental health problems. Hence, there are considerable differences in the distribution of observables between the original and estimation samples. Note, however, that we find no evidence of any selection or non-random attrition based on genetic variants, whether we use an unweighted or weighted score, or the 32 individual variants. Furthermore, there are no significant differences in children's blood pressure and fat mass between the different samples. Hence, although our analyses may be based on a selected sample of individuals, potentially limiting its generalizability, any attrition is unrelated to the genotypes used here.

### 3.2 Measures of academic achievement, blood pressure, fat mass, and the genetic variants

Our first outcome measure is the child's Key Stage 3 (KS3) score. The KS3 exam is a nationally set exam, taken by all 14-year-olds in English state schools. This measure of children's performance is therefore objective and comparable across all children. Their scores for three subjects (English, maths and science) are obtained from the National Pupil Database, a census of pupils in England in the state school system, which is matched into ALSPAC. We use an average score for the three subjects, standardized on the full sample of children for whom data is available, with mean 100, standard deviation 10.

Our second outcome measure is the child's blood pressure, measured in millimetres of mercury (mmHg) at age 13. We observe both systolic (maximum) and diastolic (minimum) blood pressure, measured by trained nurses. Blood pressure was measured twice at the clinic; we use the average of the two readings to reduce measurement error.

Our main measure for child fat mass, our risk factor of interest, is the child's body fat mass (adjusted for age in months, height and height squared), as measured by a dual-energy X-ray absorptiometry scan (DXA) at age 11. As fat mass is measured two to three years prior to the outcomes of interest, this avoids potential problems related to reverse causation. The DXA scan scans the whole body, dividing it into fat, lean tissue, and bone mass. We standardize fat mass on the full sample of children for whom data are available, with mean 100 and standard deviation 10.

For the genetic variants, we use 32 SNPs that have been consistently found to relate to body weight and fat mass (Speliotes et al., 2010; see below)^5^. There are different ways to include these instruments in our analyses, though each of these has potential drawbacks. For example, we may use the 32 variants or the 64 adiposity-increasing alleles as separate instrumental variables, potentially leading to weak instrument bias, as the effect of each individual variant or allele on adiposity is small. Alternatively, we could use a count of the total number of risk alleles carried by each individual (an ‘allelic score’), increasing the strength of the instruments. However, this imposes an equal effect size for every allele, which is not necessarily supported by the data. Similarly, we could define multiple instruments as separate dummy variables indicating the number of risk alleles carried by each individual. Although this allows for different numbers of risk alleles to have different effect sizes, it assumes that the effect of carrying e.g. four risk alleles is the same no matter which four they are. We could also use a weighted allelic score, where the weights are defined by the effect size of each particular variant on adiposity, as estimated in an independent meta-analysis.

The evidence suggests that allele scores give unbiased estimates and are more efficient than using the individual variants. There is some loss of power associated with an unweighted rather than weighted score,with the extent of the loss depending on the variation in effect sizes (see e.g. Pierce et al., 2010, Burgess and Thompson, 2013, Davies et al., 2014). Furthermore, the evidence suggests that the bias properties of the weighted and unweighted scores are robust to mis-specifications of the score, such as the presence of gene–gene and gene–environment interactions, and the mis-measurement of weights in a weighted score approach (Burgess and Thompson, 2015). In our main analysis, we use an unweighted allelic score, increasing the power of the instruments and alleviating weak IV problems. However, we also explore the alternative specifications discussed above in the sensitivity analyses.

### 3.3 Examining the validity of the genetic variants in our empirical applications

Using a total of 249,796 individuals from 64 different cohorts of European ancestry, Speliotes et al. (2010) conducted a large-scale GWAS meta-analysis. This confirmed 14 known obesity susceptibility loci and identified 18 new loci, with no evidence of non-additive effects, SNP × SNP interaction effects, or heterogeneity by sex or study. We use these 32 variants to explain variation in adiposity. We next relate the specific choice of these variants to the assumptions for suitable use of genetic variants as instruments discussed in Sections 2.2, 2.3.

Assumption 1 **Independence**

The independence assumption may be violated in the presence of population stratification due to ethnicity. For example, the allele frequencies of the SNP that accounts for the largest proportion of the variance (*FTO*) are known to vary by ethnic group (Frayling et al., 2007). Nevertheless, it is not likely to be a problem here, as our cohort is recruited from a specific geographically defined region with a predominantly white population. In addition, our analysis only includes children whose mother describes herself and the child's father as white, and we adjust all analyses for 10 ancestry-informative principal components. Furthermore, we investigate whether there is any evidence of systematic variation in the covariates by genotype. Appendix B presents the results, exploring whether the distribution of covariates is the same across the instrument distribution. Although there are no true pre-randomization variables, and significant differences do not necessarily indicate violation of independence (see also Section 2.3), we find no evidence of systematic differences for the different covariates, providing at least suggestive evidence of randomization of the genetic variants.

Assumption 2 **Exclusion**

The exclusion restriction may be violated in different situations. First, we note that those who carry the adiposity-increasing alleles of any of the variants used here do not necessarily become obese. The variants increase body weight by a modest amount, with the majority of effect sizes below 500 g. In addition, as individuals do not know their genotype, parents are unlikely to notice the subtle difference in (children's) size that is related to their genotype. Hence, it is unlikely to observe any strong responses to increased body weight, such as changing (children's) diets. However, the question remains whether there are any more subtle responses to higher body weights. As it requires large sample sizes and data on different parental preferences and behaviours to explore that in detail, one cannot say with complete certainty that behaviours are unaffected. Nevertheless, we explore this indirectly in our data, testing whether mothers’ behaviours or characteristics^6^ are related to their genotype. In the Web Appendix, Table S1, we explore whether the distribution of covariates is the same in groups defined by the *mothers*’ genotype, showing little evidence of systematic differences. This may be because there simply are no differences in maternal behaviours by genotype, because we lack sufficient power to detect any differences, or because we do not observe the behaviours that are affected by the genotype.

Second, we searched for and examine existing literature related to the mechanism through which the variants may affect fat mass. Unfortunately, little is known about the physiological function of most of our variants, with much of this work ongoing in the medical literature. Nevertheless, there is evidence that some variants (*FTO, MC4R*) are associated with an increased consumption of fat and energy (see e.g. Timpson et al., 2008, Cecil et al., 2008, Richmond and Timpson, 2012). The literature suggests that the SNPs increase food intake due to diminished satiety (Wardle et al., 2008), rather than through pathways that affect our outcomes of interest. Table S2 in the Web Appendix briefly lists what the 32 variants used here have been shown to be associated with. Although the vast majority of associations are with adiposity-related phenotypes, we note that the variants are likely to be associated with additional phenotypes not shown in the Table, either because studies that have explored these associations were underpowered, or because studies have not (yet) investigated those relationships. Depending on these additional associations, they may invalidate their use as instruments if they affect the outcome of interest directly. Nevertheless, our current knowledge suggests that, in addition to being related to adiposity and adiposity-related phenotypes (such as Type II diabetes), some variants havebeen shown to be associated with allergic asthma and rhinitis (rs10767664), hyperactivity/impulsivity (rs1307880, rs2241423), schizophrenia (rs10150332 and rs13107325), inattention (rs206936), white matter integrity (rs2815752), and Alzheimer disease risk (rs4836133, rs713586)^7^. We explore the robustness of our results to excluding these variants from the instrument setin the sensitivity analyses below.

Third, pleiotropy or LD would bias the IV estimates if the variant affects the outcome directly or if the linkage is with another variant that directly affects our outcomes of interest. We explore maps of the human genome to investigate LD, and find no evidence that the variants used here directly affect (or are in LD with variants that directly affect) our outcomes of interest or its determinants. More specifically, data from the International HapMap Project show that the SNPs that replicate in a large number of independent samples for educational attainment (Rietveld et al., 2013) and blood pressure (Ehret et al., 2011) are not in LD with those for fat mass (Speliotes et al., 2010).

Fourth, for canalization to violate the exclusion restriction, the presence of the fat related variant at conception would have to result in different brain or blood vessel development in order to counter any predicted adverse effect of fat mass on the outcome. We believe this is implausible. Furthermore, there is no evidence in humans that canalization occurs in relation to complex traits such as fat mass.

In summary, we do not know individuals’ behavioural response to their genotype, the specific physiological functions or biological pathways through which the variants affect the phenotype, and whether the variants have multiple phenotypic effects. With that, it is impossible to guarantee that Assumption 2, exclusion, holds. For instance, it is possible that some variants’ pleiotropic effects have simply not yet been identified, or that there are other variants that are in LD with our instruments that affect the outcomes of interest, but have not yet been identified. Hence, similar to any other IV approach, exclusion remains an assumption, as we cannot test for this directly.

Assumption 3 **Non-zero effect of the instrument on treatment**

The prior findings of robust associations between the genetic variants and fat mass, replicated in a large number of studies, justify their use as instruments. Using standard statistical tests, we will examine the strength of our instruments in the application below.

Assumption 4 **Monotonicity**

Finally, with random allocation of genetic variants and the fact that individuals do not know their genotypes, we assume that an individual who carries the risk allele is at least as heavy as the same individual, had she not carried the risk allele, satisfying monotonicity. Although we do not observe individuals’ counterfactuals, the literature tells us that, at a group or population level, those who possess the genetic variants are heavier than those who do not.

As monotonicity can be violated by gene–environment interactions, we examine this in two ways. First, we explore the issue of such interactions indirectly, testing whether the association between fat mass and our instrument differs in different ‘environments’. Although one can never observe all potentially relevant gene–environment interactions, we explore the importance of a set of environments that have been shown to be important for child development, defined by gender, the child's birth weight, breastfeeding duration, social class, mother's education, income and deprivation. The results (presented in Table S3 in the Web Appendix) show little evidence of gene–environment interactions.

Second, we study the existing literature on gene–environment interactions for fat mass. Kilpelainen et al. (2011) find some evidence of such interactions between *FTO* and physical activity, though only for adults: for the physically active, the *FTO* risk allele increases the odds of obesity less than for the physically inactive. They do not find such interaction for children. Using a sample of around 170,000 individuals, a recent study explores whether genetic variants explain the *variance* of BMI (as opposed to the *mean*, as gene–environment interactions by construction lead to variance inflation). They conclude that there are no common genetic variants that account for a large proportion of variation in environmental or phenotypic variability, finding no evidence of widespread gene–environment interaction effects for BMI (Yang et al., 2012).

### 3.4 Descriptive statistics

Table 2 presents the mean adiposity for each of the 32 SNPs, distinguishing between those who are homozygous for the adiposity non-increasing allele, heterozygous, and homozygous for the adiposity-increasing allele. This shows that each of the individual SNPs explain little of the variation in adiposity, leading to low power in the IV analysis. Rather than using each of the individual SNPs, we therefore use an allelic score, defined as the count of the number of adiposity-increasing alleles. As we show below, this explains a larger proportion of the variance in adiposity than the individual SNPs, increasing the power of the instrument. In the sensitivity analyses (Section 3.6), however, we also show the results using different specifications of the instrument, including the 32 individual SNPs, the 64 alleles, and a weighted allelic score.

**Fig. 1:**
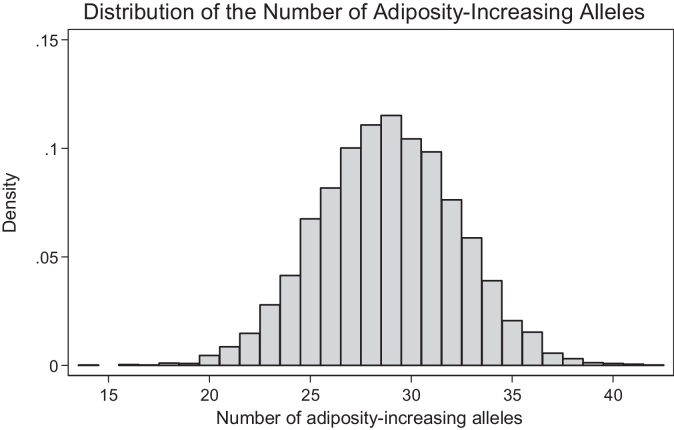
Distribution of the number of adiposity-increasing alleles carried by each child.

Fig. 1 shows a histogram of the number of adiposity-increasing alleles carried by each child, showing a bell-shaped distribution. On average, children carry 29 adiposity-increasing alleles (standard deviation = 3.4), with the total number ranging between 14 and 42.

### 3.5 Results

Table 3 presents the results. Panel A, columns 1 to 3 show the association between fat mass and the KS3 score, systolic, and diastolic blood pressure, conditional on the 10 principal components. The relationship between fat mass and educational attainment is negative, with a one standard deviation increase in fat mass associated with a 0.09 standard deviation decrease in test scores. Columns 2 and 3 show a positive association for blood pressure, with a one standard deviation increase in fat mass associated with a 0.23 and 0.12 mmHg increase in systolic and diastolic blood pressure respectively; similar to approximately 0.02 of a standard deviation.

Panel B presents the first-stage regression results, showing a strong positive relationship between the instrument and child fat mass. The strength of the relationship is shown by the first stage *F*-statistic. A value of 76 (KS3) and 69 (blood pressure) suggests the instrument is strong. Translating the effect size into body weight, a one standard deviation increase in the allelic score increases weight by around 1.3 to 1.5 kg, though the exact amount will depend on which alleles are carried by the individual, as there is much heterogeneity in the variants’ effect sizes on adiposity. For example, each risk allele of *FTO* – the variant that has been shown to have the largest effect size – increases body weight by approximately 1 kg for an average 11 year old, whereas the effect size for *GNPDA2* increases body weight by just over 300 g.

The second stage IV results are presented in Panel C. Colum 1 shows no effects of fat mass on educational performance. Although the IV estimate is of similar magnitude but opposite sign to that in Panel A, the large standard errors preclude us from rejecting the null of no effect. However, with a *p*-value of 0.054 for the Hausman test, there is some support for the IV as opposed to the OLS estimate, though any such judgement should be based on a synthesis of all the evidence, rather than on this one test alone.

Column 2 and 3 show a positive effect of fat mass on both systolic and diastolic blood pressure, with an estimate that is somewhat larger than the OLS estimates in Panel A. The estimates suggest that a one standard deviation increase in fat mass increases systolic and diastolic blood pressure by 0.37 and 0.21 mmHg respectively, though the Hausman test suggests that there is insufficient evidence to reject the hypothesis that the OLS estimate is unbiased.

### 3.6 Sensitivity analyses

We next report a set of sensitivity analyses, evaluating the robustness of the results. First, we use different specifications of the instrument. We start by specifying the 28 mutually exclusive instruments, indicating the number of adiposity-increasing alleles carried by each child. Panel A of Table 4 presents the results, showing no effect of adiposity on KS3, but a positive effect on both systolic and diastolic blood pressure. The over-identification (Hansen J) test does not reject the null. However, as discussed above, it is difficult to interpret this in a heterogeneous treatment effects framework, as a rejection may simply point to treatment effect heterogeneity.

Panel B of Table 4 uses the 32 independent genetic variants as 32 instrumental variables. Panel C specifies the number of adiposity-increasing alleles for each variant as separate instruments (i.e. 64 instruments), and Panel D uses a weighted allelic score. The latter is similar to the count of the number of risk alleles, but incorporates the fact that the variants have different effect sizes. Indeed, although the bias properties of the unweighted score have been shown to be robust to mis-specification (e.g. due to mis-measurement of weights, gene–gene or gene–environment interactions), it leads to a loss of power. We therefore use a weighted allelic score, where the weights at each locus are defined by the effect size of the variant on adiposity, estimated in an independent meta-analysis (Speliotes et al., 2010). Despite the different instrument specifications and their strength in the first stage as shown by the *F*-statistic, the results are similar.

As discussed in section 3.3, some of the variants in our analyses have been shown to associate with other, non-adiposity related, phenotypes, such as asthma and schizophrenia. In Panel E, we re-estimate the IV model with the weighted IV score that drops these variants (see Web Appendix) from the instrument set. In addition, Panel F shows the estimates using only *FTO* and *MC4R*, the two variants that account for the largest proportion of the variation in fat mass, as the instrumental variables. The estimates are similar, suggesting that the findings are not sensitive to the definition and specification of the instruments^8^.

Second, we explore the potential problem of weak instruments in more detail, using the critical values in Stock and Yogo (2005). Although the weighted and unweighted allelic scores show strong first stage results, the *F*-statistic of approximately 8, 4 and 3 in Panels A, B and C of Table 4 respectively, imply that the relative bias of the IV estimates is between 10% and 20% (Panel A) and over 30% (Panels B and C), and the size distortion over 25%. We therefore compare these estimates to different estimators that suffer less from weak instrument bias, using LIML and Fuller-*k* (with *k* *=* *1*). The critical values in Stock and Yogo (2005) show that, for example, with 27 instruments and a first stage *F*-statistic of 8.3, the size distortion in LIML is less than 10%, and Fuller has a relative bias that is less than 5%. We present the LIML and Fuller (1) results for our specification with the largest number of instruments (i.e. 64 instruments, as in Panel C of Table 4) in Table 5, showing similar estimates for the different estimators, suggesting that our results are not driven by weak instruments.

Third, we explore different definitions of the variable of interest: fat mass. As we discuss in the introduction, obese children may have different educational outcomes due to sleep disorders, school absenteeism, or differential treatment by teachers, parents and peers. This would suggest that there may be non-linearities. Hence, it is of interest to study whether there are substantially different effects on education and blood pressure for underweight and overweight children. We examine the child's underweight and overweight status in the IV analysis, defined as being in the bottom and top 15th percentile of the fat mass distribution respectively. We show the results using the (unweighted) allelic score as our instrumental variable, though they are robust to using the different instrument specifications. The results are presented in Table 6, with panel A showing the estimates of being underweight, and panel B presenting the effects for being overweight. These show that the instruments are somewhat less predictive of both binary indicators. However, with a first stage *F*-statistic ranging between 18 and 45, it is sufficiently strong, and the results show a similar pattern to those above. For Key Stage 3 (column 1), their imprecision means we cannot reject the null of no effect. For blood pressure (columns 2 and 3), the estimates confirm that being underweight substantially decreases both systolic and diastolic blood pressure, whilst being overweight leads to an increase.

Next, we explore the existence of heterogeneous effects of fat mass on KS3 and blood pressure by gender. It may be, for example, that girls are more affected by potential peers’ differential treatment related to their adiposity than boys. Table 7, Panels A and B, present the results, showing a larger (positive) effect of adiposity on educational attainment for girls than boys, though both remain insignificantly different from zero. We also find an increase in systolic blood pressure for both boys and girls, though no significant effect on diastolic blood pressure for boys. Although we are not the first to find such gender differences (see e.g. Doll et al., 2002), the reasons for the differential effects by gender are unknown, with some suggesting it may be due to gender differences in the body fat distribution (Janssen et al., 2004).

Finally, we examine the robustness of the estimates to the inclusion of different sets of covariates. As we argue in Section 2.4, our main analysis does not control for any covariates other than the 10 principal components, as any covariates are measured post-randomization and, with that, may be affected by the treatment or outcome. However, one could argue that some covariates are determined prior to, or at the time of, conception, such as child gender and some parental characteristics. Controlling for these covariates may therefore increase the precision of the estimates. Table 8 presents the results controlling for the child's gender (Panel A), for gender, maternal educational attainment, paternal social class, and the Index of Multiple Deprivation, measured at birth (Panel B), and for the full set of background characteristics mentioned in Appendix B (Panel C). We specify the (unweighted) allelic score as the instrumental variable. All findings are similar to the initial estimates: the large standard errors mean we cannot reject the null of no effect on the Key Stage 3 outcome, but we find strong positive effects on blood pressure. The confidence interval becomes slightly narrower when we control for the full set of characteristics, but it does not affect the interpretation of the findings. Taken together, these analyses show no evidence that children's fat mass affects their academic performance, but that increased fat mass leads to a rise in blood pressure.

## 4 Conclusion and discussion

Economists have become increasingly interested in the effects of behaviours such as smoking, drinking or excessive food intake on economic outcomes. As these behaviours are endogenous and difficult (if not impossible) to randomize in an RCT, estimating their effects is difficult. Many studies therefore attempt to find instrumental variables, or exploit some natural experiment that shifts the behaviour for some group, but not others, to identify their causal impact. The increasing availability of biomedical data, in combination with a growing medical literature on the effects of carrying specific genetic variants, introduces a different approach to the examination of certain risk factors on different outcomes. This paper discusses the method of instrumental variables using Mendelian randomization, and links this to the statistical potential outcomes framework. Mendelian randomization provides a novel approach to estimating the causal impact, and with that will be of interest to economists to explore.

We note that its suitability and applicability depends on a set of (biological) conditions, as well as on the research question and context. We discuss the specific conditions that need to be met for genetic variants to be used as instruments, and relate these to the statistical assumptions necessary for identification of the average causal response using instrumental variables. These conditions have not been well defined in the current social science literature, but understanding them is crucial to the appropriate use of genotypes as instruments for modifiable risk factors.

We review these conditions in the context of two empirical applications. First, we examine whether child fat mass causally affects academic achievement, and second, we study whether it affects blood pressure. To study the effect of adiposity on our outcomes of interest, a well-conducted RCT would randomize an intervention that reduces fat mass and compare the outcomes between the group that was treated and the control group. Randomizing adiposity, however, is difficult. We show that, in such cases, quasi-experimental designs such as Mendelian randomization experiments can provide an interesting alternative approach. We use a set of 32 recently identified genetic variants as instrumental variables for fat mass to illustrate the key concepts. In these illustrative examples, we show the systematic approach required to identify genetic variants as instruments. We also use direct measures of fat mass, rather than the generally used BMI. OLS shows that leaner children perform better in school tests compared to their fatter counterparts, and that fatter children have higher blood pressure. Our genetic IV analysis, however, shows no evidence that children's fat mass affects their academic performance, whilst we find that fat mass increases children's blood pressure.

Our discussion of the conditions for the suitability of genetic variants as instrumental variables and our application raise some more general issues of the use of genetic variants as instruments. First is the question whether genetic variants are powerful enough to identify causal effects. In the illustrative case we examine, while our instruments are not weak in a statistical sense their effects may be too small to impact on the possible pathways to academic performance. In other words, a 1–2 kg increase in fat mass may not lead to a large drop in self-esteem or an increase in absenteeism. It is, therefore, perhaps not surprising that we find no significant effect on academic performance. In contrast, our results on blood pressure show strong evidence of an increase driven by elevated fat mass. As intervention studies of weight reduction (see e.g. the meta-analysis of randomized controlled trials by Neter et al., 2003) have shown that a 5 kg weight loss is sufficient to cause a reduction in systolic blood pressure of about 4 mmHg, this suggests that even small changes in fat mass, such as those driven by genetic variants,can identify changes in blood pressure with sufficient precision (see also Timpson et al., 2009).

Hence, if small changes in the risk factor are sufficient to shift the outcome of interest, Mendelian randomization presents an interesting approach. However, if the outcome of interest is only affected by large changes in the risk factor, Mendelian randomization may not be sufficiently powerful, as genetic variants generally shift the risk factor by a relatively small amount. This is particularly relevant for studies in economics, which are often interested in the effects of different intermediate phenotypes (e.g. adiposity, smoking, drinking) on economic outcomes. In contrast to various examples in the medical literature, finding effects on such *economic* outcomes generally requires larger changes in the risk factor. With most genetic variants having small effect sizes, this suggests they explain insufficient variation in the risk factor to affect the outcome of interest. With a rapidly growing medical literature on the effects of carrying specific variants, one option is to wait for more variants to be identified and to combine these into one a (weighted) count of the number of risk alleles. This could increase the explained phenotypic variation and with that, the precision of the estimates. But for the type of physical attributes that economists have been interested in such as fat mass or height, any additional variants are likely to have even smaller effects than those already identified^9^.

A second issue is the credibility of the IV assumptions. Some of these are testable, but others are not. In particular, the validity of the exclusion restriction cannot be tested, and will never be known with certainty, which makes Mendelian randomization a controversial approach within the economics literature. Our paper has discussed this in detail and highlighted the various ways through which the use of genetic variants as instrumental variables may lead to invalid inferences. With our limited understanding of the functions of variants and the pathways through which they affect outcomes, it is unlikely that we will understand their exact function and mechanism in the foreseeable future. Hence, we argue that genetic variants need to be used with care. Their appropriate use requires that several conditions, which have not hitherto been spelt out in the economics literature, are met. However, even if these conditions are met, the sample sizes in data sets that contain both genetic markers and outcomes of interest to economists may be too small to obtain definitive results. Indeed, even with around 4000–5000 observations, our standard errors are relatively large. But with a rapid increase in the number of genome wide association studies, and with a decrease in their costs, this may change. Finally, just as there are good and poor RCTs and studies using any other identification design and methodology, there are good and poor Mendelian randomization studies. By highlighting the potential problems and showing the systematic approach required to identify genetic variants as instruments, we attempt to steer others to carefully consider the different assumptions and conditions needed for valid inference before jumping to their use.

## Funding:

The UK Medical Research Council (MRC), the Wellcome Trust and the University of Bristol provide core support for ALSPAC. This publication is the work of the authors, who will serve as guarantors for the contents of this paper. Funding from the Economic and Social Research Council (RES-060-23-0011 and PTA-026-27-2335), the MRC (G0601625 and G1002345), and the European Research Council (grant 269874—DEVHEALTH) is gratefully acknowledged.

## Figures and Tables

**Table B1:** An indirect test of independence: regressing the covariate on the instruments.

	Unweighted allelic score		Weighted allelic score		32 independent variants
	Coefficient	Standard error	Coefficient	Standard error	*p*-Value of *F*-test: instrument-coefficients jointly equal to zero
Girl	0.00	(0.00)	0.00	(0.01)	0.560
Birth weight (g)	2.62	(2.18)	19.22	(14.11)	0.550
Age at KS3 (in months)	0.04***	(0.02)	0.16	(0.10)	0.341
Ln(income)	0.00	(0.00)	0.00	(0.01)	0.392
Mother's education	−0.00	(0.00)	−0.00	(0.02)	0.341
Raised by natural father	0.00	(0.00)	0.00	(0.01)	0.731
Social class at birth	−0.00	(0.01)	−0.03	(0.03)	0.076
Mum works part-time	0.00	(0.00)	−0.01	(0.01)	0.971
Mum works full-time	−0.00	(0.00)	−0.00	(0.01)	0.428
Partner employed	0.00	(0.00)	0.01	(0.01)	0.905
Index of Multiple Deprivation	−0.06	(0.06)	0.32	(0.37)	0.259
Alcohol during pregnancy	0.00	(0.00)	0.01	(0.01)	0.596
Smoke during pregnancy	0.00	(0.00)	0.00	(0.01)	0.697
Breastfeeding	0.00	(0.00)	0.00	(0.03)	0.080
Mother's age at birth	0.00	(0.00)	−0.00	(0.02)	0.076

*** *p* < 0.01; The *p*-value in the last column corresponds to an *F*-test of the coefficients on the 32 instrumental variables jointly equalling zero; Ln(income) is measured when the child is aged 3–4 as is in 1995 prices; mother's educational level is a categorical variable with four values (less than ordinary (O) level, O-level, advanced (A) level, and university degree); social class is measured using the standard UK classification of class based on occupation (professional (I), managerial and technical (II), non-manual skilled (IIInm), manual skilled (IIIm), semi-skilled (IV) and unskilled (V)); the Index of Multiple Deprivation (IMD) is a measure of local area deprivation, measured at birth, referring to areas containing about 8000 persons; breastfeeding is a categorical variable (never, <1 month, 1–3 months and 3+ months); the sample contains 4846 observations.

**Table 1:** Mean and standard deviation (SD) of adiposity for each SNP.

	Original sample		Genetic sample		Estimation sample (KS3)		Estimation sample (BP)	
Outcomes	Mean	SD	Mean	SD	Mean	SD	Mean	SD
KS3	100	(10.0)	101***	(9.6)	103***	(8.9)	104***	(8.8)
Systolic BP	111	(11.5)	111	(11.3)	111	(11.2)	111	(11.2)
Diastolic BP	56.3	(8.39)	56.2	(8.28)	56.2	(8.28)	56.2	(8.28)

Treatments								
Fat mass	100	(10.0)	100	(9.8)	100	(9.8)	100	(9.7)

Instruments								
Unweighted score	28.9	(3.47)	28.9	(3.5)	28.9	(3.45)	28.9	(3.44)
Weighted score	4.02	(0.53)	4.02	(0.53)	4.02	(0.53)	4.02	(0.53)
*NRXN3*	0.42	(0.57)	0.42	(0.57)	0.42	(0.58)	0.42	(0.57)
*BDNF*	1.59	(0.58)	1.59	(0.58)	1.59	(0.58)	1.60	(0.57)
*GNPDA2*	0.87	(0.70)	0.87	(0.70)	0.86	(0.69)	0.86	(0.69)
*LRRN6C*	0.63	(0.65)	0.63	(0.65)	0.63	(0.65)	0.63	(0.65)
*PRKD1*	0.09	(0.30)	0.09	(0.30)	0.09	(0.30)	0.10	(0.30)
*GPRC5B*	1.73	(0.49)	1.73	(0.49)	1.73	(0.49)	1.73	(0.49)
*CADM2*	0.40	(0.57)	0.40	(0.57)	0.40	(0.57)	0.40	(0.57)
*SLC39A8*	0.15	(0.37)	0.15	(0.37)	0.14	(0.36)	0.15	(0.37)
*TNNI3K*	0.85	(0.70)	0.85	(0.70)	0.85	(0.70)	0.85	(0.70)
*PTBP2*	1.18	(0.70)	1.18	(0.70)	1.18	(0.70)	1.19	(0.69)
*FTO*	0.81	(0.69)	0.81	(0.69)	0.81	(0.70)	0.82	(0.70)
*NUDT3*	0.39	(0.56)	0.39	(0.56)	0.39	(0.56)	0.39	(0.56)
*FLJ35779*	1.28	(0.67)	1.28	(0.67)	1.27	(0.67)	1.27	(0.67)
*MAP2K5*	1.57	(0.58)	1.57	(0.58)	1.57	(0.58)	1.57	(0.58)
*QPCTL*	1.62	(0.56)	1.62	(0.56)	1.62	(0.56)	1.62	(0.56)
*NEGR1*	1.20	(0.69)	1.20	(0.69)	1.19	(0.69)	1.18	(0.69)
*TMEM18*	1.66	(0.53)	1.66	(0.53)	1.66	(0.53)	1.66	(0.53)
*LRP1B*	0.34	(0.53)	0.34	(0.53)	0.34	(0.53)	0.33	(0.53)
*KCTD15*	1.37	(0.66)	1.37	(0.66)	1.37	(0.65)	1.38	(0.65)
*TMEM160*	1.39	(0.64)	1.39	(0.64)	1.38	(0.64)	1.39	(0.64)
*MTCH2*	0.80	(0.69)	0.80	(0.69)	0.80	(0.70)	0.80	(0.69)
*MTIF3*	0.46	(0.59)	0.46	(0.59)	0.46	(0.59)	0.47	(0.59)
*ZNF608*	0.99	(0.71)	0.99	(0.71)	0.99	(0.71)	0.99	(0.71)
*RPL27A*	1.08	(0.70)	1.08	(0.70)	1.09	(0.70)	1.10	(0.70)
*SEC16B*	0.42	(0.58)	0.42	(0.58)	0.42	(0.58)	0.42	(0.58)
*MC4R*	0.47	(0.60)	0.47	(0.60)	0.47	(0.60)	0.47	(0.60)
*RBJ*	0.98	(0.71)	0.98	(0.71)	0.98	(0.71)	0.98	(0.71)
*FAIM2*	0.72	(0.68)	0.72	(0.68)	0.72	(0.68)	0.72	(0.67)
*SH2B1*	0.83	(0.70)	0.83	(0.70)	0.82	(0.70)	0.82	(0.70)
*FANCL*	0.58	(0.65)	0.58	(0.65)	0.58	(0.64)	0.58	(0.65)
*ETV5*	1.65	(0.54)	1.65	(0.54)	1.65	(0.53)	1.66	(0.53)
*TFAP2B*	0.36	(0.54)	0.36	(0.54)	0.36	(0.54)	0.36	(0.54)

Covariates								
Girl	0.48	(0.50)	0.49	(0.50)	0.51***	(0.50)	0.52***	(0.50)
Birth weight	3404	(557.2)	3439***	(532.1)	3436***	(525.7)	3438***	(521.6)
Age at KS3 (in months)	170	(3.73)	170	(3.77)	170	(3.68)	170	(3.71)
Ln (income)	5.29	(0.49)	5.33***	(0.48)	5.35***	(0.46)	5.37***	(0.46)
Mother's education								
O-level	0.45	(0.50)	0.44	(0.50)	0.44	(0.50)	0.44*	(0.50)
A-level	0.22	(0.42)	0.25***	(0.43)	0.26***	(0.44)	0.27***	(0.45)
University degree	0.12	(0.33)	0.15***	(0.35)	0.16***	(0.37)	0.17***	(0.38)
Not natural father	0.92	(0.28)	0.93**	(0.26)	0.93***	(0.25)	0.94***	(0.24)
Father's social class							***	
Managerial	0.34	(0.47)	0.36**	(0.48)	0.36***	(0.48)	0.37	(0.48)
Non-manual skilled	0.12	(0.33)	0.12	(0.33)	0.13*	(0.34)	0.13***	(0.34)
Manual skilled	0.31	(0.46)	0.29***	(0.45)	0.29	(0.45)	0.28***	(0.45)
Semi-skilled	0.10	(0.30)	0.09*	(0.29)	0.08***	(0.27)	0.08***	(0.27)
Unskilled	0.03	(0.17)	0.02*	(0.15)	0.02***	(0.14)	0.02***	(0.14)
Mum works part-time	0.36	(0.48)	0.38*	(0.48)	0.38***	(0.49)	0.39***	(0.49)
Mum works full-time	0.10	(0.30)	0.11	(0.31)	0.11**	(0.32)	0.11**	(0.32)
Partner employed	0.88	(0.33)	0.89***	(0.31)	0.89	(0.32)	0.89	(0.32)
IMD	20.8	(14.9)	19.7***	(14.4)	18.7***	(13.7)	18.6***	(13.6)
Alcohol in pregnancy	0.55	(0.50)	0.56	(0.50)	0.57**	(0.49)	0.58***	(0.49)
Smoke in pregnancy	0.24	(0.43)	0.21***	(0.41)	0.17***	(0.38)	0.16***	(0.37)
Breastfeed <1 month	0.16	(0.37)	0.16	(0.36)	0.16	(0.37)	0.15	(0.36)
Breastfeed 1–3 months	0.16	(0.36)	0.16	(0.36)	0.16	(0.37)	0.16	(0.37)
Breastfeed 4 + months	0.42	(0.49)	0.47***	(0.50)	0.50***	(0.50)	0.51***	(0.50)
Mother's age: 20–24	0.18	(0.39)	0.16***	(0.37)	0.13***	(0.34)	0.13***	(0.33)
Mother's age: 25–29	0.40	(0.49)	0.39	(0.49)	0.40	(0.49)	0.40	(0.49)
Mother's age:30–34	0.28	(0.45)	0.31***	(0.46)	0.33***	(0.47)	0.34***	(0.47)
Mother's age: 35+	0.10	(0.30)	0.11**	(0.31)	0.12***	(0.33)	0.12***	(0.33)
EPDS score	6.88	(4.83)	6.68***	(4.69)	6.45***	(4.56)	6.34***	(4.51)
CCEI score	13.2	(7.78)	12.8***	(7.51)	12.6***	(7.31)	12.4***	(7.22)

Sample size	11,978		7335		4844		4047	

*Notes*: Family income is an average of two observations (when the child is aged 3 and 4) and is in 1995 prices. It is adjusted for family size and composition (equalized) using the OECD equivalence scale to allow for a comparison of incomes for all households. The social class variables use the standard UK classification of social class based on occupation (professional, managerial/technical, non-manual skilled, manual skilled, semi-skilled and unskilled). IMD refers to the Index of Multiple Deprivation, and provides a relative measure of deprivation at small area level. EPDS and CCEI refer to the mother's Edinburgh Postnatal Depression Score and the Crown-Crisp Experimental Index. EPDS indicates to what extent the mother is at risk of perinatal depression; CCEI captures a broader definition of mental health, measuring general anxiety, depression and somaticism. Higher scores mean the mother is more affected. The descriptive statistics for the full sample, Column (1), are based on a maximum of 11,978 observations if the variable reported in the column has no missing values on any observations.

* *p* < 0.10,

** *p* < 0.05,

*** *p* < 0.01 refers to a test whether the mean is significantly different from the mean in the original sample shown in Column 1.

**Table 2:** Mean and standard deviation (SD) of adiposity for each SNP.

Gene	rs number	Adiposity-increasing/other allele	Homozygous for Adiposity non-increasing allele		Heterozygous		Homozygous for adiposity-increasing allele	
			Mean	SD	Mean	SD	Mean	SD
NRXN3	rs10150332	C/T	99.7	9.69	100.0	9.81	101.1	10.49
BDNF	rs10767664	A/T	99.6	10.43	99.4	9.31	100.1	9.94
GNPDA2	rs10938397	G/A	99.5	10.02	99.9	9.70	100.3	9.53
LRRN6C	rs10968576	G/A	99.9	9.89	99.8	9.67	99.8	9.66
PRKD1	rs11847697	T/C	99.7	9.68	100.9	10.66	99.4	6.81
GPRC5B	rs12444979	C/T	98.6	8.71	99.9	9.65	99.9	9.84
CADM2	rs13078807	G/A	99.6	9.61	100.3	10.01	99.6	10.27
SLC39A8	rs13107325	T/C	99.8	9.73	100.2	10.10	100.2	7.61
TNNI3 K	rs1514175	A/G	99.7	9.75	99.7	9.51	100.5	10.44
PTBP2	rs1555543	C/A	99.5	9.67	100.0	9.89	99.7	9.65
FTO	rs1558902	A/T	98.9	9.28	100.1	10.00	101.0	9.95
NUDT3	rs206936	G/A	100.0	9.77	99.4	9.70	100.0	10.31
FLJ35779	rs2112347	T/G	99.3	9.82	99.9	9.75	99.9	9.78
MAP2K5	rs2241423	G/A	99.6	9.89	99.9	10.00	99.8	9.63
QPCTL	rs2287019	C/T	99.3	8.72	100.0	9.89	99.8	9.77
NEGR1	rs2815752	A/G	99.2	9.11	100.1	9.92	99.7	9.86
TMEM18	rs2867125	C/T	98.0	9.12	99.3	9.43	100.1	9.92
LRP1B	rs2890652	C/T	99.7	9.64	100.1	10.02	99.8	10.54
KCTD15	rs29941	G/A	98.9	9.73	99.8	9.67	100.1	9.86
TMEM160	rs3810291	A/G	99.3	10.48	99.6	9.60	100.2	9.79
MTCH2	rs3817334	T/C	99.7	9.64	99.5	9.63	101.0	10.38
MTIF3	rs4771122	G/A	99.8	9.81	100.0	9.82	99.3	8.98
ZNF608	rs4836133	A/C	100.2	10.01	99.7	9.64	99.8	9.78
RPL27A	rs4929949	C/T	99.6	9.52	100.0	9.88	99.8	9.76
SEC16B	rs543874	G/A	99.5	9.55	100.3	9.99	101.5	10.86
MC4R	rs571312	A/C	99.4	9.42	100.3	10.11	101.1	10.94
RBJ	rs713586	C/T	99.1	9.98	99.8	9.63	100.7	9.76
FAIM2	rs7138803	A/G	99.6	9.67	99.9	9.87	100.6	9.72
SH2B1	rs7359397	T/C	99.6	9.70	99.8	9.83	100.2	9.76
FANCL	rs887912	T/C	100.0	9.66	99.7	9.93	99.8	9.71
ETV5	rs9816226	T/A	98.0	8.58	99.6	9.87	100.0	9.77
TFAP2B	rs987237	G/A	99.7	9.74	99.9	9.81	101.1	9.98

**Table 3:** OLS and IV estimates of the average response in standardized KS3, systolic and diastolic blood pressure.

Dependent variable	(1) KS3		(2) Systolic blood pressure		(3) Diastolic blood pressure	
	Coeff	*p*-Value	Coeff	*p*-Value	Coeff	*p*-Value
Panel A: OLS						
Fat mass coefficient and *p*-value	−0.092	*<0.001*	0.227	*<0.001*	0.116	*<0.001*
95% confidence interval	[−0.118, −0.065]		[0.188, 0.266]		[0.088, 0.144]	

Panel B: First stage IV						
Unweighted allelic score	0.349	*<0.001*	0.365	*<0.001*	0.365	*<0.001*
95% confidence interval	[0.270, 0.428]		[0.279, 0.451]		[0.279, 0.451]	

Panel C: Second stage IV						
Fat mass coefficient and *p*-value	0.114	*0.301*	0.373	*0.006*	0.205	*0.041*
95% confidence interval	[−0.102, 0.331]		[0.105, 0.642]		[0.009, 0.402]	

First stage *F*-statistic	75.47		68.78		68.78	

IV: *p*-value, Hausman test	0.054		0.276		0.365	

Number of observations	4844		4047		4047	

*Notes*: Column 1, 2 and 3 show the results, where the outcome variable is KS3, systolic and diastolic blood pressure, respectively. All analyses control for ancestry-informative principal components. 95% confidence intervals in square brackets; *p-value* is the *p*-value for standard *t*-ratio.

**Table 4:** IV robustness analyses: different specifications of the instrument.

Dependent variable	(1) KS3		(2) Systolic blood pressure		(3) Diastolic blood pressure	
	Coeff	*p*-Value	Coeff	*p*-Value	Coeff	*p*-Value
Panel A: Mutually exclusive instruments						
Coefficient and *p*-value	0.039	*0.695*	0.299	*0.015*	0.205	*0.026*
95% confidence interval	[−0.16, 0.23]		[0.06, 0.54]		[0.03, 0.39]	
First stage *F*-statistic	8.90		8.20		8.20	
IV: *p*-value, Hausman test	0.183		0.962		0.482	
IV: *p*-value, Hansen J-test	0.862		0.574		0.531	

Panel B: 32 independent SNPs						
Coefficient and *p*-value	−0.035	*0.643*	0.354	*<0.001*	0.093	*0.195*
95% confidence interval	[−0.18, 0.11]		[0.16, 0.54]		[−0.05, 0.23]	
First stage *F*-statistic	4.93		4.38		4.38	
IV: *p*-value, Hausman test	0.508		0.176		0.859	
IV: *p*-value, Hansen J-test	0.039		0.594		0.602	

Panel C: No. of adiposity-increasing alleles for each of the 32 SNPs						
Coefficient and *p*-value	−0.031	*0.647*	0.372	*<0.001*	0.168	*0.011*
95% confidence interval	[−0.16, 0.10]		[0.20, 0.55]		[0.04, 0.30]	
First stage *F*-statistic	3.11		2.63		2.63	
IV: *p*-value, Hausman test	0.277		0.082		0.267	
IV: *p*-value, Hansen J-test	0.422		0.531		0.144	

Panel D: Weighted allelic score						
Coefficient and *p*-value	0.080	*0.370*	0.452	*<0.001*	0.174	*0.038*
95% confidence interval	[−0.10, 0.26]		[0.23, 0.68]		[0.01, 0.34]	
First stage *F*-statistic	116.7		101.3		101.3	
IV: *p*-value, Hausman test	0.049		0.045		0.482	
IV: *p*-value, Hansen J-test	–		–		–	

Panel E: Selected instruments in weighted score						
Coefficient and *p*-value	0.116	*0.239*	0.542	*<0.001*	0.182	*0.037*
95% confidence interval	[−0.08, 0.31]		[0.30, 0.78]		[0.01, 0.35]	
First stage *F*-statistic	96.59		91.68		91.68	
IV: *p*-value, Hausman test	0.029		0.444		0.444	
IV: *p*-value, Hansen J-test	–		–		–	

Panel F: Using only *FTO* and *MC4R*						
Coefficient and *p*-value	0.139	*0.312*	0.666	*<0.001*	0.230	*0.082*
95% confidence interval	[−0.13, 0.41]		[0.30, 1.03]		[−0.03, 0.49]	
First stage *F*-statistic	24.54		21.81		21.81	
IV: *p*-value, Hausman test	0.078		0.011		0.340	
IV: *p*-value, Hansen J-test	0.403		0.192		0.147	

Number of observations	4844		4047		4047	

*Notes*: See notes to Table 3. Panel A shows the IV estimate using the mutually exclusive instrumental variables, indicating the number of adiposity-increasing alleles carried by each child. Panel B uses the 32 independent SNPs as 32 instruments. Panel C specifies the number of adiposity-increasing alleles for each SNP as separate instruments (i.e. 64 instruments). Panel D uses a weighted allelic score, where the weights at each locus are defined by the effect size of the variant on adiposity, as estimated in an independent meta-analysis (Speliotes et al., 2010). All analyses control for ancestry-informative principal components. 95% confidence intervals in square brackets; *p-value* is *p*-value for standard *t*-ratio.

**Table 5:** IV robustness analyses: LIML and Fuller(1).

Dependent variable	(1) KS3		(2) Systolic blood pressure		(3) Diastolic blood pressure	
	Coeff	*p*-Value	Coeff	*p*-Value	Coeff	*p*-Value
Panel A: LIML						
Fat mass coefficient and *p*-value	−0.001	*0.991*	0.477	*0.002*	0.216	*0.084*
95% confidence interval	[−0.196, 0.194]		[0.179, 0.775]		[−0.029, 0.462]	
First stage *F*-statistic	3.11		2.63		2.63	
*p*-value, Hausman test	0.277		0.082		0.267	
*p*-value, Hansen J-test	0.434		0.608		0.156	

Panel B: Fuller(1)						
Coefficient and *p*-value	−0.002	*0.985*	0.474	*0.002*	0.215	*0.082*
95% confidence interval	[−0.195, 0.192]		[0.180, 0.769]		[−0.027, 0.458]	
First stage *F*-statistic	3.11		2.63		2.63	
*p*-value, Hausman test	0.277		0.082		0.267	
*p*-value, Hansen J-test	0.433		0.605		0.156	
						
Number of observations	4844		4047		4047	

*Notes*: The models include 64 instruments, indicating whether the child carries each of the adiposity-increasing alleles for each SNP (as in Panel C of Table 4). All analyses control for ancestry-informative principal components. See also notes to Table 3.

**Table 6:** IV robustness analyses: different specifications of the variable of interest.

Dependent variable	(1) KS3		(2) Systolic blood pressure		(3) Diastolic blood pressure	
	Coeff	*p*-Value	Coeff	*p*-Value	Coeff	*p*-Value
Panel A: Pr(Underweight)						
Coefficient and *p*-value	−5.861	*0.315*	−19.26	*0.021*	−10.56	*0.065*
95% confidence interval	[−17.3, 5.6]		[−35.66, −2.85]		[−21.77, 0.64]	
First stage *F*-statistic	19.91		18.22		18.22	
*p*-value, Hausman test	0.205		0.012		0.060	

Panel B: Pr(Overweight)						
Coefficient and *p*-value	3.916	*0.310*	13.31	*0.009*	7.117	*0.044*
95% confidence interval	[−3.64, 11.47]		[3.46, 22.49]		[0.21, 14.03]	
First stage *F*-statistic	45.49		40.24		40.24	
*p*-value, Hausman test	0.106		0.139		0.242	
Number of observations	4844		4047		4047	

*Notes*: See notes to Table 3. Panel A presents the estimates for the effect of being underweight; Panel B shows the estimates for being overweight.

**Table 7:** IV robustness analyses: subgroup analysis.

Dependent variable	(1) KS3		(2) Systolic blood pressure		(3) Diastolic blood pressure	
	Coeff	*p*-Value	Coeff	*p*-Value	Coeff	*p*-Value
Panel A: Girls						
Coefficient and *p*-value	0.243	*0.106*	0.426	*0.035*	0.447	*0.004*
95% confidence interval	[−0.05, 0.54]		[0.03, 0.82]		[0.14, 0.75]	
First stage *F*-statistic	43.43		33.65		33.65	
*p*-value, Hausman test	0.008		0.263		0.020	
Number of observations	2469		2089		2089	

Panel B: Boys						
Coefficient and *p*-value	−0.029	*0.866*	0.329	*0.088*	−0.043	*0.767*
95% confidence interval	[−0.37, 0.31]		[−0.05, 0.71]		[−0.33, 0.24]	
First stage *F*-statistic	32.62		34.51		34.51	
*p*-value, Hausman test	0.736		0.738		0.228	
Number of observations	2375		1958		1958	

*Notes*: See notes to Table 3. Panels A and B present the estimates for girls and boys respectively.

**Table 8:** IV robustness analyses: controlling for covariates.

	(1) KS3		(2) Systolic blood pressure		(3) Diastolic blood pressure	
	Coeff	*p*-Value	Coeff	*p*-Value	Coeff	*p*-Value
Panel A: Covariates: gender						
Fat mass coefficient and *p*-value	0.111	*0.319*	0.379	*0.006*	0.206	*0.042*
95% confidence interval	[−0.11, 0.33]		[0.11, 0.65]		[0.01, 0.40]	
First stage *F*-statistic	77.18		69.51		69.51	
*p*-value, Hausman test	0.044		0.288		0.371	

Panel B: Covariates: gender, maternal education, social class at birth, IMD						
Fat mass coefficient and *p*-value	0.092	*0.342*	0.379	*0.007*	0.206	*0.046*
95% confidence interval	[−0.10, 0.28]		[0.10, 0.66]		[0.00, 0.41]	
First stage *F*-statistic	79.61		68.61		68.61	
*p*-value, Hausman test	0.122		0.288		0.363	

Panel C: Covariates: all^1^						
Fat mass coefficient and *p*-value	0.090	*0.322*	0.374	*0.008*	0.201	*0.052*
95% confidence interval	[−0.09, 0.27]		[0.10, 0.65]		[−0.00, 0.40]	
First stage *F*-statistic	81.56		69.35		69.35	
*p*-value, Hausman test	0.156		0.284		0.382	

*N*	4844		4047		4047	

*Notes*: See notes to Table 3. All analyses control for ancestry-informative principal components.

1 All covariates includes: child birth weight, age at KS3 (in months), ln(income), mother's educational attainment, a binary indicator whether the child was raised by the natural father, social class at birth, dummy variables indicating whether the mother works part-time or full-time, and whether the partner is employed, the Index of Multiple Deprivation (IMD, measured at birth), binary variables indicating whether the mother drank alcohol during pregnancy, and whether the mother smoked during pregnancy, the duration of breastfeeding, and mother's age at birth. The definition of the covariates is given in the note to Table B1, Appendix B.

## Appendix A A brief introduction to genetics

Each cell in the human body contains a nucleus in which most DNA (99.9995%) is kept. DNA is stored in structures called chromosomes, where each chromosome contains a single continuous piece of DNA. All cells in the human body apart from germ cells contain 46 chromosomes, organized into 23 chromosome pairs: one copy of chromosome 1–22 from each parent, plus an X-chromosome from the mother and either an X or a Y chromosome from the father.

Locations (or loci) where DNA varies between people are called polymorphisms. The most commonly studied form of polymorphism is a Single Nucleotide Polymorphism (SNP): a single base-pair variation in a DNA locus. As chromosomes come in pairs, humans have two base-pairs at each locus, called alleles. These alleles can either be the same or different. The term genotype is used to describe the specific set of alleles inherited at a particular chromosome locus. For example, individuals can have one of three genotypes of *FTO*: they can be homozygous for the common allele (TT), heterozygous (AT), and homozygous for the rare allele of *FTO* (AA). The visible or measurable effect of a particular genotype is called the phenotype. The phenotype, or risk factor, we examine is fat mass.

Many studies have examined the heritability of fat mass, defined as the proportion of the total variance that is explained by genetic factors. These are most commonly calculated from twin studies, comparing intra-pair correlations for a characteristic in monozygotic twins with that in dizygotic twins. These studies generally report large heritability estimates: between 0.4 and 0.7. A high heritability however, does not imply that any individual genetic variant has large phenotypic effects. For example, there are many SNPs that affect human weight, though all with small effects: so-called ‘polygenes’. Together, these variants may have a large phenotypic effect.

Until recently, researchers mainly used a ‘candidate gene approach’ to examine associations between individual genetic variants and a risk factor. This approach consists of testing a specific hypothesis: based on biological knowledge, researchers examine the association between one particular variant (the candidate genetic variant) and a risk factor. These studies produced many false-positive findings (Colhoun et al., 2003) and were inefficient. Genome wide association studies (GWAS) followed, genotyping 500,000 to over 1,000,000 SNPs in one go and relating all SNPs to the risk factorof interest in a hypothesis-free way. Stringent criteria are used for GWAS *p*-values to take account of this hypothesis-free approach. Studies are either two-stage studies, where one or more GWAS is performed, after which the small number of SNPs that reach GWAS levels of statistical significance are typed in other independent samples to examine the robustness. Alternatively, studies consist of a number of independent GWAS containing a large total sample size, where only those SNPs that have consistent associations across all studies are interpreted as robust.

## Appendix B Indirect test of independence of covariates and genetic variants

Table B1 presents the coefficients (standard errors) of a regression of the covariate in the first column on the unweighted allelic score (columns 1 and 2) or weighted allelic score (columns 3 and 4). The final column shows the *p*-value of an *F*-statistic testing whether the coefficients on the 32 independent variants jointly equal zero. With random assignment of the genetic variants, there should be no systematic variation in the covariates by genotype. Although some coefficients are significantly different from zero, there is no evidence of systematic differences for the different covariates, providing at least suggestive evidence of randomization of genetic variants.

## Footnotes

1 Appendix A provides a brief guide to the terms used in genetic studies.

2 Thus we give below examples of situations where the use of genetic variants as instrumental variables is *more* as well as *less* likely to lead to incorrect inference.

3 Note that not all combinations of rare alleles may be observed in the data, reducing the total number of instruments. In addition, when multiple pairs of risk alleles give the same predicted treatment, one may condense the number of instrument values, leading to a further reduction in the total number of instruments (see Clarke et al., 2015).

4 For more information on the representativeness of ALSPAC, its enrolment and response rates, see Boyd et al., 2012, Fraser et al., 2012; and www.bristol.ac.uk/alspac.

5 We discuss these in more detail below, as well as in the Web Appendix. In short, however, these are *NRXN3* (rs10150332), *BDNF* (rs10767664), *GNPDA2* (rs10938397), *LRRN6C* (rs10968576), *PRKD1* (rs11847697), *GPRC5B* (rs12444979), *CADM2* (rs13078807), *SLC39A8* (rs13107325), *TNNI3K* (rs1514175), *PTBP2* (rs1555543), *FTO* (rs1558902), *NUDT3* (rs206936), *FLJ35779* (rs2112347), *MAP2K5* (rs2241423), *QPCTL* (rs2287019), *NEGR1* (rs2815752), *TMEM18* (rs2867125), *LRP1B* (rs2890652), *KCTD15* (rs29941), *TMEM160* (rs3810291), *MTCH2* (rs3817334), *MTIF3* (rs4771122), *ZNF608* (rs4836133), *RPL27A* (rs4929949), *SEC16B* (rs543874), *MC4R* (rs571312), *RBJ* (rs713586), *FAIM2* (rs7138803), *SH2B1* (rs7359397), *FANCL* (rs887912), *ETV5* (rs9816226), *TFAP2B* (rs987237), where the rs-number is an identification tag that uniquely positions the polymorphism in the genome.

6 Including her education, age, lone parenthood, employment, smoking, drinking, duration of breastfeeding, locus of control, a ‘teaching score’, and two scores indicating her engagement with and interest in outdoor, and in indoor activities with the child.

7 The associations with hyperactivity, impulsivity, and inattention were not replicable in independent samples. For more information, see the Web Appendix.

8 The results in Panel E and F that use a restricted instrument set are also robust to using an unweighted score, or using the variants/alleles separately as instruments.

9 For example, the most recent (at time of writing) GWAS identified an additional 56 BMI-associated loci, which generally have lower minor allele frequency and/or smaller effect size estimates than previously known loci (Locke et al., 2015).
