# Supplementary material for: Genetic markers as instrumental variables
Source: J Health Econ. 2016 Jan;45:131–48. doi: 10.1016/j.jhealeco.2015.10.007 (PMC4770870; doi:10.1016/j.jhealeco.2015.10.007)
Supplement: Supplementary file 1 [file mmc1.docx]

# Web Appendix: Supplemental Information

1. Maternal behaviours, characteristics, and genotypes

Table S1 below indirectly examines whether mothers’ behaviours or characteristics are related to their genotype. It presents the coefficients (standard errors) of a regression of the covariate (i.e. the maternal behaviour / characteristic) in the first column on the unweighted *maternal* allelic score (columns 1 and 2) or weighted maternal allelic score (columns 3 and 4). The final column shows the *p*-value of an *F*-statistic testing whether the coefficients on the 32 independent *maternal* variants jointly equal zero.

With random assignment of the genetic variants, there should be no systematic variation in the characteristics and behaviours by genotype. We find some significant differences. For example, more risk alleles are associated with a higher probability that the mother is employed part-time. However, there is little evidence of any *systematic* differences for the different covariates shown here.

Table S1: An indirect test of independence: regressing the covariate on the *maternal* genetic variants

|  | Unweighted allelic score | | Weighted allelic score | | 32 independent variants |
| --- | --- | --- | --- | --- | --- |
|  | Coefficient | Standard error | Coefficient | Standard error | *p*-value of F-test: instrument-coefficients jointly equal to zero |
| Mother’s education | -0.00 | (0.00) | -0.02 | (0.02) | 0.172 |
| Mother’s age at birth | -0.00 | (0.00) | -0.04 | (0.03) | 0.076 |
| Raised by natural father | -0.00 | (0.00) | -0.00 | (0.01) | 0.275 |
| Mum works part-time | 0.00** | (0.00) | 0.02* | (0.01) | 0.097 |
| Mum works full-time | 0.00 | (0.00) | 0.00 | (0.01) | 0.371 |
| Alcohol during pregnancy | 0.00 | (0.00) | 0.03* | (0.01) | 0.402 |
| Smoke during pregnancy | 0.00 | (0.00) | 0.01 | (0.01) | 0.270 |
| Breastfeeding | -0.00 | (0.01) | 0.01 | (0.03) | 0.040 |
| Mother’s locus of control | 0.02 | (0.04) | 0.31 | (0.27) | 0.319 |
| Mother’s teaching score | 0.00 | (0.00) | 0.02 | (0.03) | 0.139 |
| Mother’s engagement with child’s activities | 0.00 | (0.00) | 0.00 | (0.01) | 0.258 |
| Parental engagement in active play with child | 0.00 | (0.02) | 0.04 | (0.13) | 0.098 |

Notes: * p<0.1; ** p<0.05; *** p<0.01; The *p*-value in the last column corresponds to an *F*-test of the coefficients on the 32 maternal instrumental variables jointly equalling zero. Mother’s educational level is a categorical variable with four values (less than ordinary O-level, O-level, A-level, and university degree). Breastfeeding is a categorical variable (never, <1 month, 1-3 months and 3+ months). Locus of control is a psychological concept that describes whether individuals attribute successes and failures to internal or external causes. Those with an internal (low) locus of control see themselves as responsible for the outcomes of their actions; those with an external (high) locus of control believe that successes and failures are chance-determined. Mother’s teaching score is constructed from questions that measure whether the mother is involved in teaching her child (depending on the child’s age) songs, the alphabet, being polite, etc. We use an average score from three measures at ages 18, 30 and 42 months to capture longer-term involvement. The mother’s engagement with the child’s activities measures the extent to which the mother reads/sings to the child, helps the child build towers, etc. Parental engagement with active play is a continuous indicator measuring the extent to which the parents engage in active (outdoor) activities with their children, such as going to the park, going swimming, etc. The sample contains 4,925 observations.

1. The 32 Genetic Variants

Table S2 shows the 32 genetic variants used in our analysis, and briefly lists what the variant has been shown to be associated with. The majority of the information below is obtained from the GWAS catalogue^[[1]](#footnote-1)^, and [www.SNPedia.com](http://www.SNPedia.com). However, we note that the genetic variants are very likely to be associated with additional phenotypes not listed here, either because studies that have explored these associations were underpowered, or because studies have not (yet) investigated that specific relationship.

Wherever possible, we also discuss what is known about the variant’s mechanism. However, as very little is known about the function of specific genes, this information is limited. For example, even for the *FTO* genetic variant, the one that accounts for the largest proportion of the variation in fat mass (and with that, the one studied most extensively, as it is the easiest to identify), the physiological function is unknown, with the current evidence only suggesting that it affects obesity through increased consumption of fat and energy, and diminished satiety. As there are – in some cases – hundreds of studies showing the association between the genetic variant and body mass index or obesity (for the largest GWAS, see Speliotes et al., (2010)), Table S2 only cites studies that find associations with outcomes other than adiposity-related phenotypes.

Table S2: Known functions of the 32 genetic variants used in our analyses

| rs number | Gene | Associations: |
| --- | --- | --- |
| rs10150332 | NRXN3 | Shown to be associated with body mass index, obesity, and fat distribution (waist to hip ratio). One study finds an association between *NRXN3* and schizophrenia (Wang et al., 2014). |
| rs10767664 | BDNF | *BDNF* codes for proteins that are responsible for maintaining energy balance through food consumption and energy expenditure (Garver et al., 2013). rs10767664 has also been associated with allergic asthma (Andiappan et al, 2011) and allergic rhinitis (Jin et al., 2015). Furthermore, the BDNF gene has been implicated in learning and memory among rodents (Cunha et al., 2010) |
| rs10938397 | GNPDA2 | Shown to be related to body mass index, fat mass percentage, waist circumference, waist-to-height ratio, obesity and other health states associated with obesity, such as type II diabetes and cardiovascular risk (He et al., 2010; Xi et al., 2013) |
| rs10968576 | LRRN6C | Shown to be associated with body mass index and obesity |
| rs11847697 | PRKD1 | Shown to be associated with body mass index and obesity |
| rs12444979 | GPRC5B | Shown to be associated with body mass index and obesity |
| rs13078807 | CADM2 | Shown to be associated with body mass index and obesity. One study also shows an association between rs13078807 and hyperactivity/impulsivity, but could not replicate this in an independent sample (Albayrak et al., 2013) |
| rs13107325 | SLC39A8 | Shown to be associated with body mass index, obesity. It is also related to blood pressure and HDL cholesterol, outcomes associated with obesity, and likely on the causal pathway. This SNP has also been associated with schizophrenia (Carrera et al., 2012) |
| rs1514175 | TNNI3K | Shown to be associated with body mass index and obesity |
| rs1555543 | PTBP2 | Shown to be associated with body mass index and obesity |
| rs1558902 | FTO | Shown to be associated with body mass index, obesity, waist circumference, weight, and metabolic syndrome. It is also related to other outcomes associated with obesity, such as type II diabetes, and age at menarche, and asthma. It is expressed in the hypothalamus and functionally involved in energy homeostasis. The SNP is believed to affect obesity through an increased consumption of fat and energy (Timpson et al., 2008, Cecil et al., 2008, Richmond and Timpson, 2012), as well as due to diminished satiety (Wardle et al., 2008) |
| rs206936 | NUDT3 | Shown to be associated with body mass index and obesity. One study also shows an association between rs206936 and inattention, but could not replicate this in their meta-analysis (Albayrak et al., 2013) |
| rs2112347 | FLJ35779 | Shown to be associated with body mass index and obesity |
| rs2241423 | MAP2K5 | Shown to be associated with body mass index and obesity. One study shows an association between rs2241423 and hyperactivity, but could not replicate this in their meta-analysis (Albayrak et al., 2013) |
| rs2287019 | QPCTL | Shown to be associated with body mass index, obesity and the insulinogentic index (for the latter, see Burgdorf et al., 2012) |
| rs2815752 | NEGR1 | Shown to be associated with body mass index and obesity. One study also shows an association between rs2815752 and white matter integrity (Dennis et al., 2014) |
| rs2867125 | TMEM18 | Shown to be associated with body mass index and obesity |
| rs2890652 | LRP1B | Shown to be associated with body mass index, obesity and insulin resistance (for the latter, see Burgdorf et al., 2012) |
| rs29941 | KCTD15 | Shown to be associated with body mass index, obesity, metabolic syndrome, and colorectal cancer risk, though the association with colorectal cancer risk turned insignificant after adjustment for multiple comparisons (for the latter, see Lim et al., 2012) |
| rs3810291 | TMEM160 | Shown to be associated with body mass index and obesity |
| rs3817334 | MTCH2 | Shown to be associated with body mass index and obesity |
| rs4771122 | MTIF3 | Shown to be associated with body mass index and obesity |
| rs4836133 | ZNF608 | Shown to be associated with body mass index and obesity. One study also shows an association with Alzheimer’s disease risk (for the latter, see Hinney et al., 2014) |
| rs4929949 | RPL27A | Shown to be associated with body mass index and obesity |
| rs543874 | SEC16B | Shown to be associated with body mass index and obesity. It is also related to the age at menarche, an outcome associated with obesity |
| rs571312 | MC4R | Shown to be associated with body mass index, obesity, and colorectal cancer risk, though the association with colorectal cancer risk turned insignificant after adjustment for multiple comparisons (for the latter, see Lim et al., 2012). *MC4R* codes for proteins that are responsible for maintaining energy balance through food consumption and energy expenditure (Garver et al., 2013). Like *FTO*, it is associated with an increased consumption of fat and energy and decreased energy expenditure |
| rs713586 | RBJ | Shown to be associated with body mass index and obesity. One study also shows an association with Alzheimer’s disease risk (for the latter, see Hinney et al., 2014) |
| rs7138803 | FAIM2 | Shown to be associated with body mass index, obesity, fat mas percentage, waist circumference, weight, metabolic syndrome, and waist-to-height ratio |
| rs7359397 | SH2B1 | Shown to be associated with body mass index and obesity, total fat, waist circumference, serum leptin (Jamshidi et al., 2007), and insulin sensitivity (Fall et al., 2012) |
| rs887912 | FANCL | Shown to be associated with body mass index and obesity |
| rs9816226 | ETV5 | Shown to be associated with body mass index and obesity |
| rs987237 | TFAP2B | Shown to be associated with body mass index, obesity, and adiposity |

1. Gene-environment interactions

Monotonicity can be violated by gene-environment interactions. We explore the existence of such interactions indirectly, by testing whether the association between fat mass and the allelic score differs in different ‘environments’. Although one can never observe all potentially relevant environments, we explore the importance of a set of environments that have been shown to be important for child development. In particular, we investigate whether the effect of the allelic score differs by (1) the child’s gender, (2) birth weight, (3) breastfeeding duration, (4) social class at the child’s birth, (5) maternal education, (6) family income, and (7) deprivation. Specifically, we interact the allelic score with each of these environments in a regression of fat mass on the interactions (i.e. the first stage). We then test whether the interaction-coefficients can be statistically distinguished in different environments.

Table S3 presents the results. This shows that the effect of the allelic score on adiposity differs slightly in different environments. For example, one additional risk allele increases adiposity in boys by 0.33 (about 3% of a standard deviation), and in girls with 0.36. However, we cannot statistically distinguish between these estimates (*p = 0.70*). Similarly, there is little evidence of gene-environment interactions in the analyses presented in columns 2 to 7.

Table S3. Gene-environment interactions

|  | (1)  Gender | (2)  Birth weight | (3)  Duration of breastfeeding | (4)  Social class at birth | (5)  Maternal education | (6)  Family income | (7)  Index of Multiple Deprivation |
| --- | --- | --- | --- | --- | --- | --- | --- |
|  |  |  |  |  |  |  |  |
| Allelic score, group 1 | 0.36*** | 0.36*** | 0.37*** | 0.38*** | 0.37*** | 0.28*** | 0.27*** |
|  | (0.06) | (0.08) | (0.10) | (0.06) | (0.11) | (0.08) | (0.08) |
| Allelic score, group 2 | 0.33*** | 0.38*** | 0.25*** | 0.31*** | 0.39*** | 0.42*** | 0.38*** |
|  | (0.06) | (0.08) | (0.10) | (0.06) | (0.06) | (0.08) | (0.08) |
| Allelic score, group 3 |  | 0.36*** | 0.37*** | 0.40*** | 0.34*** | 0.42*** | 0.35*** |
|  |  | (0.08) | (0.10) | (0.13) | (0.08) | (0.08) | (0.08) |
| Allelic score, group 4 |  | 0.30*** | 0.37*** |  | 0.21*** | 0.28*** | 0.44*** |
|  |  | (0.08) | (0.06) |  | (0.10) | (0.08) | (0.08) |
|  |  |  |  |  |  |  |  |
| *p-*value testing equality of *G*E* | *0.70* | *0.91* | *0.74* | *0.68* | *0.48* | *0.40* | *0.56* |
| Number of observations | 4844 | 4844 | 4844 | 4844 | 4844 | 4844 | 4844 |

Notes: The estimates in each column are obtained from one regression. This is a regression of fat mass on the allelic score, the ‘environment’ indicated in the column heading (e.g. gender, birth weight), and their interaction, as well as the 10 ancestry-informative principal components. For column 1, group 1 and 2 indicate girls and boys respectively. In column 2, groups 1 to 4 indicate the quartiles of the birth weight distribution. The duration of breastfeeding is grouped as no breastfeeding (group 1), less than 1 month (group 2), between 1-3 months (group 3), and 4 or more months (group 4). Social class at birth (column 4) is subdivided into professional/managerial/technical (group 1), non-manual and manual skilled (group 2), and semi-skilled/unskilled (group 3). Column 5, maternal education, indicates less than O-level (group1), O-level (group 2), A-level (group 3), and university degree (group 4). In columns 6 and 7, groups 1 to 4 indicate the quartiles of the income and IMD distribution respectively. The “*p-*value testing equality of *G*E*” is the *p*-value of an *F-*test testing whether the coefficient on the different groups are significantly different from each other.

**References**

Albayrak, O., et al., 2013. Common obesity risk alleles in childhood attention-deficit/hyperactivity disorder. *American Journal of Medical Genetics. Part B*. 162B, 295-305.

Andiappan, A., et al., 2011. Genetic variation in BDNF is associated with allergic asthma and allergic rhinitis in an ethnic Chinese population in Singapore. *Cytokine*, 56, 218-23.

Burgdorf, K., et al. 2012. Association Studies of Novel Obesity-related Gene Variants with Quantitative Metabolic Phenotypes in a Population-Based Samples of 6,039 Danish Individuals. Diabetologia, 55, 105-13.

Carrera, N., et al., 2012. Association Study of Nonsynonymous Single Nucleotide Polymorphisms in Schizophrenia. *Biological Psychiatry*, 71, 169-77.

Cecil, J., Tavendale, R., Watt, P., et al. (2008) An obesity-associated FTO gene variant and increased energy intake in children. *New England Journal of Medicine,* 359, 2558-2566.

Cunha, C. et al., 2010. A simple role for BDNF in learning and memory? *Froniers in Molecular Neuroscience*, 3, 1-14.

Dennis, E., et al.,m 2914. Obesity Gene NEGR1 assocaited with White Matter Integrity in Healthy Young Adults. *Neuroimage*, 15, 548-57.

Fall, T., et al., 2012. The role of obesity-related genetic loci in insulin sensitivity. Diabetic Medicine, 29, e62-6.

Garver et al., 2013. The Genetics of Childhood Obesity and Interaction with Dietary Macronutrients. *Genes and Nutrition*, 8, 271-87.

He, M., et al., 2010. Obesity genotype score and cardiovascular risk in women with type 2 diabetes mellitus. *Arteriosclerosis, Thrombosis, and Vascular Biology*, 30, 327-32.

Hinney et al., 2014. Genetic variation at the CELF1 (CUGBP, elav-like family member 1 gene) locus is genome-wide associated with Alzheimer’s disease and obesity. *American Journal of Medical Genetics. Part B*. 165B, 283-93

Jamshidi et al., 2007. The SH2B gene is associated with serum leptin and body fat in normal female twins. Obesity, 15, 5-9.

Jin, P., et al., 2015. A functional brain-derived neurotrophic factor (BDNF) gene variant increases the risk of moderate-to-severe allergtic rhinitis. *The Journal of Allergy and Clinical Immunology*, 135, 1486-93.

Lim et al., 2012. Susceptibility Variants for obesity and colorectal cancer risk: The multiethnic cohort and PAGE studies. *International Journal of Cancer*, 131, E1038-43.

Richmond, R. and Timpson, N. 2012. Recent Findings on the Genetics of Obesity: Is there a Public Health Relevance? *Current Nutrition Reports*, 1, 239-248.

Speliotes, E.K., et al. (2010) Association analyses of 249,796 individuals reveal 18 new loci associated with body mass index. *Nature Genetics,* 42(11):937-950.

Timpson, N.J., Emmett, P.M., Frayling, T.M., et al. (2008) The Fat Mass- and Obesity-Associated Locus and Dietary Intake in Children. *The American Journal of Clinical Nutrition,* 88, 971-978.

Wang et al., 2014. A genome wide association study on obesity and obesity-related traits. In: Obesity Epidemiology, Pathogenesis and Treatment: A Multidisciplinary Approach (Ed: R. Ahima) Taylor & Francis Group.

Wardle, J., Carnell, S., Haworth, C.M.A. et al (**2008) Obesity Associated Genetic Variation in *FTO* is associated with Diminished Satiety. *The Journal of Clinical Endocrinology and Metabolism,* 93, 3640-3643.**

Xi, B. et al., 2013. Associations of obesity susceptibility loci with hypertension in Chinese children. *International Journal of Obesity*, 37, 926-930.

1. Available at: [https://www.genome.gov/page.cfm?pageid=26525384&clearquery=1#searchForm](https://www.genome.gov/page.cfm?pageid=26525384&clearquery=1#searchForm) [↑](#footnote-ref-1)
